# Patient Reported Outcomes and Complications of Stress Incontinence Surgery: Effect of Patient Characteristics

**DOI:** 10.1007/s00192-025-06507-1

**Published:** 2026-01-14

**Authors:** Fiona Bach, Christina Easter, Alice Sitch, Katie Morris, Philip Toozs-Hobson

**Affiliations:** 1 Christchurch Women’s Hospital, 2 Riccarton Avenue, Christchurch, 8011 New Zealand; 2 Department of Applied Health Sciences, University of Birmingham, National Institute for Health and Care Research (NIHR) Birmingham Biomedical Research Centre, Birmingham, UK; 3 Institute of Applied Health Research, University of Birmingham, Birmingham, UK; 4 Birmingham Clinical Trials Unit, University Hospitals Birmingham NHS Foundation Trust and University of Birmingham, Birmingham, UK; 5 Maternal Fetal Medicine, Birmingham Women’s and Children’s Hospital, Birmingham, UK; 6 NHS West Midlands, Birmingham Women’s Hospital, University of Birmingham, Birmingham, UK

**Keywords:** Database, Patient-reported outcomes, Personalised medicine, Stress urinary incontinence surgery, Surgical outcome, Surgical complications

## Abstract

**Introduction and Hypothesis:**

Multiple procedures exist to treat stress urinary incontinence. A database records outcomes and complications and ascertains how surgical and patient characteristics affect outcomes.

**Methods:**

A retrospective cohort study of 31,901 women undergoing continence surgery from the British Society of Urogynaecologists Surgical Database (2008–2019), including 24,923 retropubic mesh-tapes, 4740 bulking agents, 538 fascial slings and 1700 colposuspension. Multivariable logistic regression was used for primary analyses to compare outcomes between treatments and secondary analysis to assess how different characteristics affect outcomes within treatment groups.

**Results:**

Similar outcomes for patient reported global impression of improvement were observed following retropubic mesh-tapes, fascial slings and colposuspension (91%, 89%, 87%, respectively) compared to bulking agents (56.6%). For retropubic mesh-tapes, reduced odds of positive global impression of improvement was seen with increased age, body mass index, detrusor overactivity and intraoperative bladder injury. Odds of bladder injury increased with non-consultant grade operator and decreased with increasing BMI. For colposuspension, increased age led to decreased odds of success and increased odds of return to hospital and readmission. Repeat procedures led to decreased odds of success for retropubic tapes, bulking agents and colposuspension.

**Conclusions:**

This large national database demonstrated that increased age, higher BMI, preoperative detrusor overactivity and bladder injury are associated with treatment failure. This information should be used in bespoke counselling to encourage personalised medical decision-making. Missing data is a limitation and would be improved with a mandatory database.

**Supplementary Information:**

The online version contains supplementary material available at 10.1007/s00192-025-06507-1.

## Introduction

Stress urinary incontinence (SUI) is a bothersome condition seen in up to 34% of women in the United Kingdom (UK) [1]. Management options are varied and have undergone significant changes in both technique, and physician and patient preference, over the past 40 years. Conservative measures include fluid management, weight loss [2], eliminating chronic cough, pelvic floor muscle training (PFMT) [3], vaginal pessary [4, 5], and pharmacological treatment [6, 7]. Surgical options had been dominated by retropubic mesh tapes (RPT) [8–12], with periurethral bulking agents (PUB) [13–15], autologous fascial sling (AFS) [16, 17], and colposuspension [18, 19] less commonly used [20]. The mesh “pause” mandated by the UK government following the Cumberlege report [21] has led to removal of mesh products from the market initiating renewed interest in other techniques. There is therefore the need for up-to-date surgical outcome data on these re-emerging techniques in current clinical practice. Postoperative outcomes can be affected by a variety of patient [20, 22–34] and surgical factors [35]. Identification of the impact of these factors may allow patients to make a more informed, bespoke choice of surgical procedure. Patients can choose to defer intervention whilst undertaking lifestyle changes to modify characteristics that could compromise outcomes.

The British Society of Urogynaecology Surgical (BSUG) Database allows clinicians to record details of incontinence and prolapse operations. It is voluntary, non-mandatory, and recommended by several bodies, including NICE [6] and NHS England [36–38]. It is widely used with over 250 registered users in the UK. Preoperative patient characteristics, surgical details and complications are entered following the procedure, with outcomes and postoperative complications recorded at follow-up [39], between 6 weeks to 12 months according to clinician practice. The BSUG database can only be accessed on NHS (N3 connected) computers. It is password protected and is accessed by web browser and runs on a 256 AES SSL connection. Security is run by Daisy Communications (IGSoC and ISO27001 accredited). All data are encrypted to NHS standards.

The primary objective of this study was to determine and compare the patient reported outcomes and complications following RPT, PUB, AFS and colposuspension for patients entered on the BSUG database. Furthermore, the secondary objective was to ascertain whether any surgical or patient characteristics entered onto the database affected these outcomes. This will aim to help determine the “right” treatment in terms of patient choice, suitability and efficacy depending on individual patient characteristics.

## Methods

### Acquiring the Data

This was a retrospective cohort study, performed on a dataset sourced from the BSUG database for female patients undergoing surgery for SUI from January 2008 to March 2019. Patients are entered by the operating surgeon at the time of surgery and outcomes are entered when patients are seen for postoperative review. Data were acquired following a two-stage process, initially approved by the BSUG Research Committee and subsequently ratified by the BSUG Database Committee. Anonymisation of data prior to release ensured individual patients, doctors and surgical centres could not be identified. Individual centres using the database are required to be compliant with UK Caldicott Principles [40] and General Data Protection Regulations (GDPR) and patients are required to consent to data being entered on the database, including its use for research.

### Study Population

Patients from the database were included if they underwent RPT, PUB, AFS and colposuspension (open, laparoscopic) in the UK under NHS or private care. The primary population was defined as patients who were having their first SUI surgery without other concomitant major surgery. The secondary population was all patients from eligible centres to allow comparison of first and repeat surgery.

### Classification and Grouping of Procedures

Patients were included if they underwent RPT using Advantage, Advantage Fit and TVT and for bulking agents; Bulkamid, Collagen, Contigen, Macroplastique and Tegress were used. All fascial sling techniques were included as the Aldridge style and sling on a string (SOAS) have been found to be equally as effective [41]. Open and laparoscopic colposuspension were merged, as similar success rates with both techniques are seen [42, 43]. Patients were included if the centre they were operated at reported ≥ 10 incontinence procedures in any given year to reflect centres that reported regularly rather than on an ad hoc basis.

For analysis, a concomitant surgery was classified as a prolapse surgery or a hysterectomy performed at the same time. A hysteroscopy, cystoscopy or laparoscopy without hysterectomy was not deemed significant enough to alter the outcomes studied.

### Outcome Measures

Outcome measures were already set by the database committee when the database was first set up.

The primary outcome was the validated Patient Reported Global Impression of Improvement (PGII) [44]. PGII is a Likert scale of 7 points which was categorised into two groups for analysis defined as “better” incorporating very much better and much better and “not better” incorporating a little better, no change, a little worse, much worse and very much worse [45].

Secondary outcomes included change in SUI symptoms and change in overactive bladder (OAB) symptoms. The database records whether the symptoms of SUI and OAB were cured, improved, no change, worse, a new symptom or never present, which have been shown to relate well to symptom improvement, based on Medical Epidemiologic and Social Aspects of Ageing (MESA) scores [46, 47]. For SUI; “cured” or “improved” counted as success compared to “no change” or “worse”. If patients had preoperative OAB symptoms their options for reporting would be cured, improved, no change or worse. If the patient reported “new OAB“ or “OAB never present” this, by default, meant they did not have OAB prior to the procedure. Other secondary outcomes were bladder injury, return to theatre within 72 h, return to hospital within 30 days and readmission to hospital within 30 days.

### Statistical Analysis

Baseline measures were summarised as frequency and percentages for categorical variables and mean and standard deviation or median and interquartile ranges for continuous measures where appropriate.

Multivariable logistic regression was used. The model for the primary analysis included all treatments in the primary population to allow comparisons across all four procedures, with RPT being designated the reference treatment. The secondary analysis included each procedure in separate models to identify characteristics that altered the outcome of that procedure. Odds ratios (OR), 95% confidence intervals and *p* values were reported for all analyses.

Clinically relevant covariates [48–50] included in the models were age, BMI, PFMT, preoperative UDS, grade of operator, bladder injury and whether the surgery was the first SUI surgery or a repeat. Missing data was addressed by applying the technique of multiple imputation using chained equations for each of the treatment datasets separately, between 63 and 81 imputations were used on the basis of the fraction of missing data, with imputations combined using Rubin’s rules [51, 52]. Analysis of the data was performed using Stata v16 (StataCorp College Station, USA).

### Sample Size Calculations

There was no prospective sample size calculation as all available data was used from the on-going database. However, to identify a difference of 5% (90% vs 85%) in the primary outcome of PGII between groups would require 918 participants per group (1836 total number of participants) to achieve 90% power at a significance level of 5%. As there were 10,084 women receiving RPT and 1273 women receiving PUB, there was sufficient power to compare these groups for the primary outcome in the primary population.

## Results

**Fig. 1:**
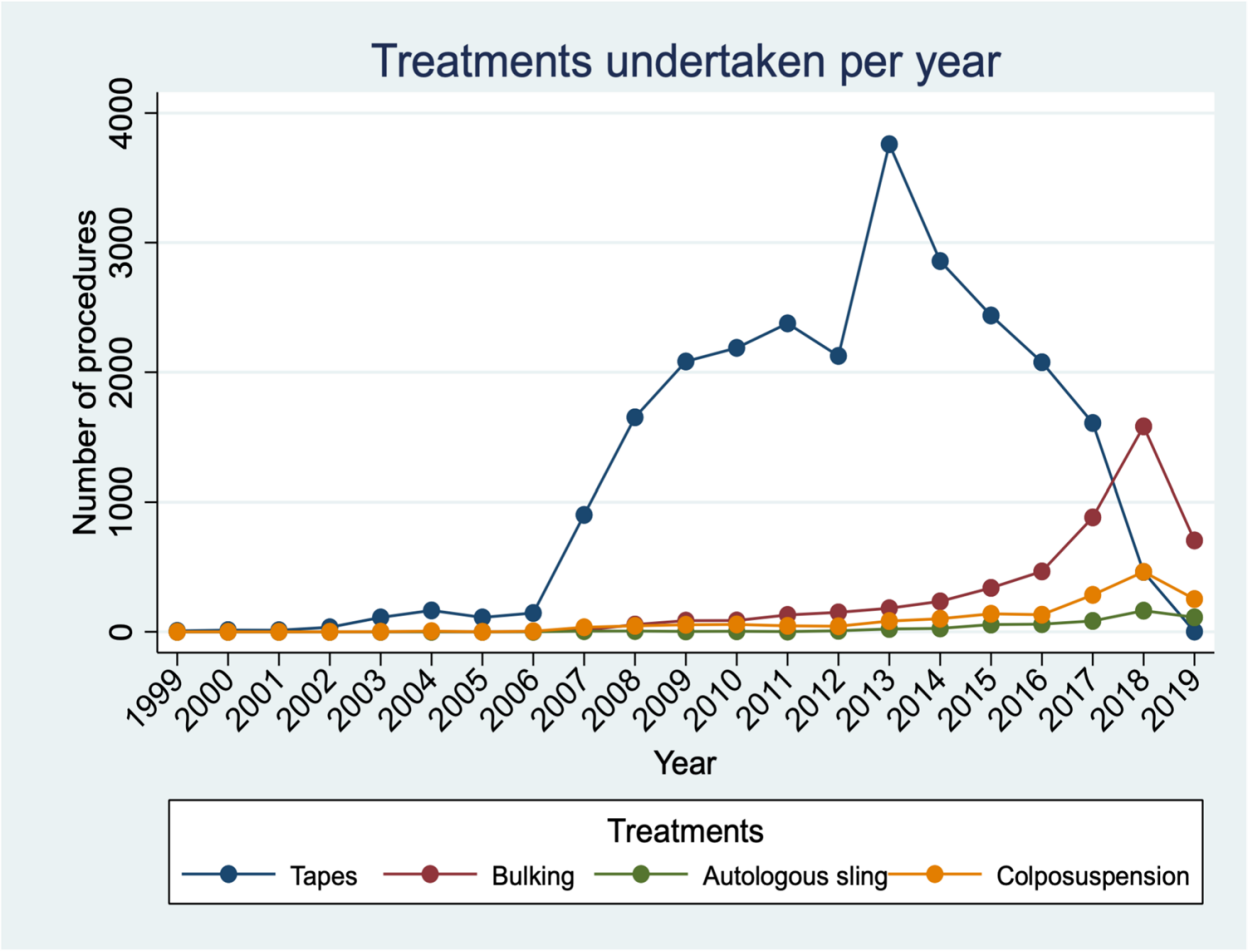
Number of procedures entered onto the BSUG database per year (NB Data were retrieved in March in the final year so for that year there was only a quarter of the projected numbers of procedures. Data could be added retrospectively starting in 1999)

There were 31,901 procedures available for analysis. RPT was the most common procedure performed (*n* = 24,923; 78.1%), then PUB (*n* = 4740; 14.8%), colposuspension (*n* = 1700; 5.3%) and AFS (*n* = 538; 1.7%). Figure 1 shows how the recorded treatments changed over time. Mesh procedures fell in 2017, and PUB became rapidly more popular with a slower increase in colposuspension and AFS. In 2013 HQIPP (Healthcare Quality Improvement Partnership) undertook a national audit and this explains the increase in the procedures recorded (rather than a change in practice).

### Patient Characteristics

For the primary population, the mean ages of patient receiving RPT, AFS and colposuspension were similar (between 48 and 51 years with around 11 years standard deviation (SD)). PUB demonstrated a slightly higher mean age at 55 years (15 years SD). A greater proportion of patients receiving PUB were over 80 years (7.4%, 166/2237) compared to RPT (1.5%, 230/15478), AFS (0.6%, 1/171) and colposuspension (0.4%, 3/697). The mean BMI of all procedures were similar across all procedures (around 29 with a SD of 5). Between 90 and 92% of patients were documented to have had PFMT across all procedures. Consultants performed the majority of these procedures (between 78 and 88%). The most common urodynamic diagnosis was urodynamic stress incontinence (USI) for all procedures (between 75 and 86%). All baseline characteristics are summarised in Table 1.

**Table 1:** Baseline characteristics for the primary population

Treatment	RPT	PUB	AFS	Colposuspension
*N* = 19,106	*n* = 15,885	*n* = 2316	*n* = 177	*n* = 728
Age (years) mean (SD)	51.66 (11.48)	55.31 (15.61)	48.55 (11.04)	49.32 (10.66)
missing, *n*	407	79	6	31
Age (years) categories, *n* (%)				
Age less than 50	7621 (49.2%)	909 (40.6%)	98 (57.3%)	367 (52.7%)
Age between 50 and 79	7627 (49.3%)	1162 (51.9%)	72 (42.1%)	327 (46.9%)
Age 80 and over	230 (1.5%)	166 (7.4%)	1 (0.6%)	3 (0.4%)
Missing, *n*	407	79	6	31
BMI, mean (SD)	28.38 (4.94)	29.06 (5.40)	28.29 (4.55)	28.53 (4.77)
Missing, *n*	8340	1596	113	401
Pelvic floor exercises, *n* (%)				
Yes	12,888 (90.9%)	1863 (90.9%)	148 (92.5%)	600 (90.1%)
No	1288 (9.1%)	186 (9.1%)	12 (7.5%)	66 (9.9%)
Missing, *n*	1709	267	17	62
Grade of operator, *n* (%)				
Consultant	12,299 (78.3%)	2003 (88.2%)	140 (82.4%)	608 (84.1%)
Non consultant	3404 (21.7%)	267 (11.8%)	30 (17.6%)	115 (15.9%)
Missing, *n*	182	46	7	5
Pre-op urodynamic diagnosis, *n* (%)				
USI, USI + voiding dysfunction	11,626 (77.9%)	1623 (75.5%)	129 (77.2%)	579 (86.3%)
DOA, mixed, mixed + voiding dysfunction	2421 (16.2%)	403 (18.7%)	27 (16.2%)	77 (11.5%)
Normal, voiding dysfunction, not done	877 (5.9%)	124 (5.8%)	11 (6.6%)	15 (2.2%)
Missing, *n*	961	166	10	57

For the secondary population, 94.2% (22,742/24153) of RPT were the first SUI surgery compared to 73.5% for PUB (3220/4380), 57.1% for AFS (285/499) and 78.8% for colposuspension (1248/1584). The characteristics of the secondary population were similar to the primary population (Appendix 1).

### Primary Analysis: Comparison Across Treatments: Primary Population: Primary Outcome

For patients in the primary population with a reported primary outcome, 91.0% recorded a “better” outcome for PGII following RPT (9175/10084), 89.1% for AFS (57/64) and 87.4% for colposuspension (353/404) with a lower proportion for PUB of 56.6% (721/1273) (Table 2). Multivariable analysis showed an 87% reduction in the odds of a positive score for PGII following PUB compared to RPT which is statistically significant (OR 0.13; 95% CI 0.09,0.17; *p* < 0.001) (Table 3). When evaluating the PGII outcome between AFS and colposuspension compared to RPT, there were no significant differences (OR 1.12 (0.25, 4.93) and OR 0.96 (0.56, 1.65), respectively).

**Table 2:** Patient reported outcomes and complications for the primary population with non-concomitant first surgery

Treatment	RPT *n* = 15,885		PUB *n* = 2316		AFS *n* = 177		Colposus-pension *n* = 728		Total
PGII, *n* (%)									
Better	9175	91.0%	721	56.6%	57	89.1%	353	87.4%	
Not better	909	9.0%	552	43.4%	7	10.9%	51	12.6%	
Total	10,084		1273		64		404		11,825
*Missing PGII data*	*5801*		*1043*		*113*		*324*		
SUI symptoms, *n* (%)									
Cured or improved	9131	96.7%	874	74.1%	59	96.7%	376	96.7%	
No change or worse	312	3.3%	305	25.9%	2	3.3%	13	3.3%	
Total	9443		1179		61		389		11,072
*Missing SUI data*	*6442*		*1137*		*116*		*339*		
OAB									
OAB (Patients with preoperative symptoms), *n* (%)									
Cured or improved	3432	62.4%	260	38.6%	12	36.4%	102	45.9%	
No change or worse	2067	37.6%	413	61.4%	21	63.6%	120	54.1%	
Total	5499		673		33		222		6,427
OAB (Patients without preoperative symptoms), *n* (%)									
New symptoms	378	10.0%	10	2.4%	4	16.0%	22	14.6%	
Never present	3406	90.0%	404	97.6%	21	84.0%	129	85.4%	
Total	3784		414		25		151		4,374
*Missing OAB data*	*6602*		*1229*		*119*		*355*		
Bladder injury, *n* (%)									
Yes	569	3.6%	2	0.1%	6	3.4%	24	3.3%	
No	15,123	96.4%	2292	99.9%	169	96.6%	698	96.7%	
Total	15,692		2294		175		722		18,883
*Missing*	*193*		*22*		*2*		*6*		
Return to theatre for procedure-related event within 72 h, *n* (%)									
Yes	59	0.5%	0	0.0%	0	0.0%	2	0.4%	
No	11,454	99.5%	1418	100%	72	100%	461	99.6%	
Total	11,513		1418		72		463		13,466
*Missing*	*4372*		*898*		*105*		*265*		
Return to hospital within 30 days for procedure related event, *n* (%)									
Yes	231	6.8%	22	1.9%	12	18.5%	27	7.1%	
No	3146	93.2%	1137	98.1%	53	81.5%	353	92.9%	
Total	3377		1159		65		380		4981
*Missing*	*12,508*		*1157*		*112*		*348*		
Readmitted to hospital within 30 days for procedure related event, *n* (%)									
Yes	430	3.8%	10	0.7%	11	15.1%	25	5.5%	
No	10,867	96.2%	1392	99.3%	62	84.9%	428	94.5%	
Total	11,297		1402		73		453		13,225
*Missing*	*4588*		*914*		*104*		*275*

**Table 3:** Univariable and multivariable comparison of outcomes for the primary analysis of comparisons across treatment for the primary population

		Result compared to RPT univariable		Result compared to RPT multivariable	
Outcome	Procedure	OR (95% CI)	*p* value	OR (95% CI)	*p* value
PGII	PUB	0.10 (0.09, 0.12)	< 0.001*	0.13 (0.09,0.17)	< 0.001*
	AFS	0.69 (0.30, 1.55)	0.367	1.12 (0.25,4.93)	0.882
	Colposuspension	0.67 (0.48, 0.92)	0.014*	0.96 (0.56,1.65)	0.874
SUI symptoms	PUB	0.08 (0.06,0.09)	< 0.001*	0.10 (0.06,0.14)	< 0.001*
	AFS	0.89 (0.21,3.76)	0.874	0.26 (0.06,1.17)	0.079
	Colposuspension	0.95 (0.53,1.71)	0.859	1.04 (0.40,2.72)	0.933
OAB symptoms (pre-op OAB)	PUB	0.59 (0.48,0.73)	< 0.001*	1.24 (0.86,1.80)	0.254
	AFS	0.46 (0.21,1.00)	0.051	0.61 (0.12,3.06)	0.550
	Colposuspension	0.94 (0.68,1.29)	0.681	0.99 (0.60,1.65)	0.970
OAB symptoms (no pre-op OAB)	PUB	0.59 (0.48, 0.73)	< 0.001	0.20 (0.05,0.84)	0.028*
	AFS	0.46 (0.21, 1.00)	0.051	2.53 (0.51,12.68)	0.259
	Colposuspension	0.94 (0.68,1.29)	0.681	1.11 (0.46,2.66)	0.822
Bladder injury	PUB	0.02 (0.01,0.10)	< 0.001*	0.03 (0.00,0.19)	< 0.001*
	AFS	0.71 (0.29,1.73)	0.449	1.45 (0.50,4.25)	0.496
	Colposuspension	0.80 (0.51,1.27)	0.346	0.92 (0.50,1.68)	0.776
Return to theatre		unable to complete as no returns to theatre for PUB or AFS			
Return to hospital (RTH) within 30 days	PUB	0.25 (0.16,0.40)	< 0.001*	0.32 (0.19,0.53)	< 0.001*
	AFS	2.52 (1.24,5.10)	0.010*	2.42 (1.07,5.45)	0.033*
	Colposuspension	1.01 (0.64,1.59)	0.964	1.05 (0.62,1.78)	0.848
Readmitted to hospital (RdTH)	PUB	0.20 (0.11,0.39)	< 0.001*	0.22 (0.10,0.47)	< 0.001*
	AFS	4.84 (2.43,9.65)	< 0.001*	5.81 (2.64,12.75)	< 0.001*
	Colposuspension	1.44 (0.93,2.25)	0.104	1.56 (0.93,2.61)	0.092

The multivariable analysis adjusted for the following: age, BMI, PFMT, pre-op UDS, grade of operator and bladder injury

### Primary Analysis: Comparison Across Treatments: Primary Population: Secondary Outcomes

For patients in the primary population, generally secondary outcomes appeared to differ only when comparing PUB to RPT. Similar results were seen when comparing AFS and colposuspension with RPT with the exceptions of readmission and return to hospital (Table 3).

Cure/improvement of SUI was seen in 96.7% (9175/10084) of patients after RPT, 96.7% (59/61) after AFS and 96.7% (376/389) after colposuspension with a lower percentage for PUB of 74.1% (874/1179) (Table 2) resulting in a statistically significant reduction in odds of a positive outcome for PUB compared to RPT (OR 0.10; 95% CI 0.06,0.14; *p* < 0.001) (Table 3).

For those patients with preoperative OAB symptoms, RPT showed the highest percentage of cure/improvement of OAB of 62.4% (3432/5499) compared to 38.6% (260/673) for PUB, 36.4% (12/33) with AFS and 45.9% (102/222) with colposuspension with no statistically significant findings (Table 2).

For those patients without preoperative OAB, new OAB was reported by 16% (4/25) of AFS and 14.6% (22/151) of colposuspension patients compared to 10% (378/3784) for RPT and a lower percentage for PUB (2.4% (10/414). There was a reduction in the odds of new OAB being reported for those with PUB (OR 0.20; 95% CI 0.05,0.84) compared to RPT. For those with existing OAB, PUB and RPT were similar (OR 1.24 (0.86, 1.80)).

The percentage of patients who experienced a bladder injury was similar for RPT (3.6% 569/15692), AFS (3.4% 6/175) and colposuspension (3.3% 24/722) (Table 2) with a lower odds for PUB (0.1% 6/175) (OR 0.03; 95% CI 0.00,0.19) (Table 3) compared to RPT.

Return to theatre percentage was similar for RPT (0.5% 59/11454) and colposuspension (0.4% 2/463) with no returns to theatre seen with PUB and AFS (Table 2). Return to hospital was recorded for 6.8% (231/3377) of RPT patients compared to 1.9% with PUB (22/1159) (OR 0.32; 95% CI 0.19,0.53). For AFS return to hospital was recorded for 18.5% of patients (12/65) (OR 2.42; 95% CI 1.07,5,45) and 7.1% for colposuspension (27/380) (OR 1.05 (0.62, 1.78)). Readmission to hospital was recorded for 3.8% (430/10867) of RPT patients compared to 0.7% (10/1402) for PUB (OR 0.22; 95% CI 0.10,0.47), and higher for AFS with 15.1% (11/73) readmitted (OR 5.81; 95% CI 2.64,12.75) and colposuspension with 5.5% readmitted (25/453) (OR 1.56 (0.93, 2.61) (Tables 2 and 3). The reasons for return to hospital included trial without catheter, voiding difficulties, infection, active bleeding and haematoma.

### Secondary Analysis: Comparison Within Treatments: The Effect of Patient and Operative Characteristics: Primary Population

For RPT, the odds of a positive score of PGII reduced with increased age (OR 0.83; 95% CI 0.77,0.90; *p* < 0.001), increased BMI (OR 0.95; 95% CI 0.93,0.97; *p* < 0.001), preoperative UDS that included overactivity (OR 0.51; 95% CI 0.41,0.64; *p* < 0.001) and intraoperative bladder injury (OR 0.57; 95% CI 0.37,0.89; *p* = 0.012) (Table 4). The same characteristics were also found to reduce the odds of a positive outcome for symptoms of SUI following an RPT: increased age (OR 0.81; 95% CI 0.72,0.91; *p* < 0.001), increased BMI (OR 0.96; 95% CI 0.93,0.99; *p* = 0.009), preoperative UDS that included overactivity (OR 0.65; 95% CI 0.45,0.93; *p* = 0.017) and bladder injury (OR 0.38; 95% CI 0.21,0.71; *p* = 0.002) (Table 4). Following an RPT, greater odds of developing new postoperative OAB were observed with increased age (OR 1.20; 95% CI 1.06, 1.36; *p* = 0.003) and non-consultant grade operator (OR 1.5; 95% CI 1.07,2.09; *p* = 0.018). Odds of bladder injury following an RPT increased with a non-consultant grade operator (OR 3.88; 95% CI 2.99, 5.05) and decreased with increasing BMI (OR 0.93; 95% CI 0.90,0.96). Increased odds of return to theatre following RPT was seen with non-consultant grade (OR 3.81; 95% CI 1.96,7.41; *p* < 0.001) and bladder injury (OR 4.23; 95% CI 1.81,9.90; *p* = 0.001). Bladder injury during RPT was the sole factor associated with increased odds of readmission to hospital (OR 2.10; 95% CI 1.23,3.58; *p* = 0.006).

**Table 4:** Multivariable comparison of outcomes for the primary analysis separately for treatments for the primary population: characteristics in the primary population that lead to a change in the odds of success for RPT, PUB, AFS and colposuspension on multivariable analysis

Outcome measure	Characteristics		RPT		PUB		AFS		Colposuspension	
			OR (95% CI)	*p* value	OR (95% CI)	*p* value	OR (95% CI)	*p* value	OR (95% CI)	*p* value
PGII	Age (decades)		0.83 (0.77,0.90)	< 0.001	1.04 (0.95,1.14)	0.425	0.47 (0.10,2.32)	0.354	0.82 (0.59,1.12)	0.215
	BMI		0.95 (0.93,0.97)	< 0.001	0.98 (0.95,1.02)	0.341	0.66 (0.24,1.87)	0.435	0.98 (0.89,1.08)	0.714
	PFMT		1.11 (0.84,1.47)	0.472	1.04 (0.63,1.72)	0.880	-	-	1.00 (0.34,2.93)	0.999
	UDS	USI, USI and voiding dysfunction	Reference		Reference		Reference		Reference	
		DOA, Mixed, mixed + voiding dysfunction	0.51 (0.41,0.64)	< 0.001	1.11 (0.78,1.58)	0.572	1.23 (0.03,52.24)	0.914	2.81 (0.81,9.69)	0.102
		Normal, voiding dysfunction, not done	0.92 (0.60,1.43)	0.718	0.51 (0.26,0.99)	0.047	0.09 (0.00,20.21)	0.388	-	-
	Grade of operator	Consultant	Reference		Reference		Reference		Reference	
		Other	0.85 (0.68,1.06)	0.146	0.71 (0.46,1.09)	0.117	0.01 (0.00,551.52)	0.410	0.53 (0.21,1.30)	0.167
	Bladder injury		0.57 (0.37,0.89)	0.012	-		-	-	0.56 (0.10,3.04)	0.498
SUI	Age (decades)		0.81 (0.72,0.91)	< 0.001	0.93 (0.83,1.04)	0.180	2.97 (0.04,214.36)	0.615	0.47 (0.25,0.87)	0.017
	BMI		0.96 (0.93,0.99)	0.009	0.99 (0.95,1.03)	0.468	0.60 (0.19,1.92)	0.380	0.94 (0.76,1.15)	0.536
	PFMT		1.04 (0.66,1.62)	0.866	1.17 (0.66,2.07)	0.599	-	-	1.13 (0.13,9.93)	0.914
	UDS	USI, USI and voiding dysfunction	Reference		Reference		Reference		Reference	
		DOA, Mixed, mixed + voiding dysfunction	0.65 (0.45,0.93)	0.017	1.02 (0.67,1.56)	0.910	-	-	-	-
		Normal, voiding dysfunction, not done	0.96 (0.47,1.94)	0.900	0.59 (0.29,1.22)	0.156	0.07 (0.00,2041.87)	0.616	-	-
	Grade of operator	Consultant	Reference		Reference		Reference		Reference	
		Other	0.72 (0.51,1.03)	0.069	0.96 (0.57,1.63)	0.890	0.00 (0.00,366.06)	0.287	0.56 (0.10,3.16)	0.514
	Bladder injury		0.38 (0.21,0.71)	0.002	-		-	-	0.40 (0.04,4.10)	0.438
OAB – previous OAB symptom	Age (decades)		0.98 (0.92,1.04)	0.511	1.01 (0.89,1.15)	0.846	4.93 (0.02,1114.89)	0.564	1.20 (0.86,1.68)	0.289
	BMI		0.99 (0.97,1.00)	0.092	1.01 (0.97,1.06)	0.665	1.19 (0.37,3.84)	0.766	1.01 (0.92,1.11)	0.813
	PFMT		0.87 (0.67,1.15)	0.335	0.98 (0.51,1.90)	0.955	-	-	0.64 (0.21,2.03)	0.453
	UDS	USI, USI and voiding dysfunction	Reference		Reference		Reference		Reference	
		DOA, Mixed, mixed + voiding dysfunction	0.92 (0.76,1.11)	0.386	1.37 (0.89,2.11)	0.157	0.05 (0.00,7689.26)	0.617	1.57 (0.70,3.50)	0.274
		Normal, voiding dysfunction, not done	1.43 (0.93,2.20)	0.102	0.91 (0.33,2.48)	0.851	-	-	-	-
	Grade of operator	Consultant	Reference		Reference		Reference		Reference	
		Other	0.88 (0.73,1.07)	0.216	0.58 (0.30,1.13)	0.109	-	0.999	0.57 (0.17,1.96)	0.372
	Bladder injury		0.92 (0.60,1.41)	0.696	-		-	-	1.29 (0.18,9.11)	0.799
OAB – no previous OAB	Age (decades)		1.20 (1.06,1.36)	0.003	1.19 (0.69,2.04)	0.528	1.14 (0.08,17.08)	0.916	0.89 (0.52,1.51)	0.657
	BMI		1.01 (0.98,1.04)	0.534	1.11 (0.94,1.30)	0.219	0.99 (0.75,1.32)	0.965	1.04 (0.89,1.23)	0.599
	PFMT		1.01 (0.62,1.65)	0.975	-	-	-	-	0.88 (0.21,3.69)	0.857
	UDS	USI, USI and voiding dysfunction	Reference		Reference		Reference		Reference	
		DOA, Mixed, mixed + voiding dysfunction	0.96 (0.54,1.72)	0.900	-	-	8.27 (0.28,247.51)	0.223	2.16 (0.27,17.09)	0.467
		Normal, voiding dysfunction, not done	0.86 (0.49,1.53)	0.615	-	-	-	-	1.93 (0.12,31.03)	0.641
	Grade of operator	Consultant	Reference		Reference		Reference		Reference	
		Other	1.50 (1.07,2.09)	0.018	1.52 (0.17,13.81)	0.710	-	-	1.45 (0.35,5.98)	0.606
	Bladder injury		1.18 (0.60,2.31)	0.639	-		-	-	4.93 (0.24,102.60)	0.303
Bladder injury	Age (decades)		1.10 (0.99,1.22)	0.081	-	-	0.08 (0.00,4.09)	0.208	1.67 (1.03,2.71)	0.036
	BMI		0.93 (0.90,0.96)	< 0.001	-	-	0.88 (0.43,1.80)	0.729	1.01 (0.89,1.15)	0.867
	UDS	USI, USI and voiding dysfunction	Reference		Reference		Reference		Reference	
		DOA, Mixed, mixed + voiding dysfunction	0.75 (0.51,1.10)	0.140	-	-	-	-	1.64 (0.43,6.23)	0.465
		Normal, voiding dysfunction, not done	0.58 (0.31,1.10)	0.095	-	-	-	-	-	-
	Grade of operator	Consultant	Reference		Reference		Reference		Reference	
		Other	3.88 (2.99,5.05)	< 0.001	-	-	0.36 (0.00,1171.97)	0.802	1.00 (0.21,4.74)	0.998
Return to theatre within 72 h	Age (decades)		0.90 (0.68,1.19)	0.461	Nil	Nil	Nil	Nil	1.26 (0.13,12.07)	0.843
	BMI		0.98 (0.91,1.05)	0.530	Nil	Nil	Nil	Nil	1.01 (0.41,2.46)	0.984
	UDS	USI, USI and voiding dysfunction	Reference		Reference		Reference		Reference	
		DOA, Mixed, mixed + voiding dysfunction	0.48 (0.17,1.42)	0.185	Nil	Nil	Nil	Nil	-	-
		Normal, voiding dysfunction, not done	1.45 (0.42,5.09)	0.558	Nil	Nil	Nil	Nil	-	-
	Grade of operator	Consultant	Reference		Reference		Reference		Reference	
		Other	3.81 (1.96,7.41)	< 0.001	Nil	Nil	Nil	Nil	-	-
	Bladder injury		4.23 (1.81,9.90)	0.001	-		Nil	Nil	-	-
Return to hospital within 30 days	Age (decades)		1.00 (0.86,1.15)	0.956	1.26 (0.91,1.75)	0.168	0.95 (0.53,1.71)	0.867	2.03 (1.31,3.16)	0.002
	BMI		0.99 (0.95,1.03)	0.594	1.04 (0.92,1.17)	0.513	0.85 (0.68,1.07)	0.162	0.96 (0.84,1.10)	0.575
	UDS	USI, USI and voiding dysfunction	Reference		Reference		Reference		Reference	
		DOA, Mixed, mixed + voiding dysfunction	0.85 (0.54,1.33)	0.469	2.63 (0.82,8.49)	0.106	0.41 (0.04,3.96)	0.444	0.50 (0.10,2.46)	0.394
		Normal, voiding dysfunction, not done	1.34 (0.70,2.57)	0.378	4.02 (1.02,15.90)	0.047	-	-	-	-
	Grade of operator	Consultant	Reference		Reference		Reference		Reference	
		Other	1.25 (0.84,1.86)	0.270	3.87 (0.90,16.58)	0.068	-	-	0.57 (0.10,3.11)	0.517
	Bladder injury		1.70 (0.85,3.42)	0.134	-		-	-	1.56 (0.15,16.09)	0.711
Readmission to hospital within 30 days	Age (decades)		1.05 (0.94,1.17)	0.357	1.10 (0.65,1.87)	0.723	2.03 (1.10,3.77)	0.024	1.82 (1.12,2.95)	0.016
	BMI		1.00 (0.98,1.03)	0.794	0.96 (0.77,1.19)	0.710	1.07 (0.85,1.34)	0.572	1.10 (0.96,1.26)	0.163
	UDS	USI, USI and voiding dysfunction	Reference		Reference		Reference		Reference	
		DOA, Mixed, mixed + voiding dysfunction	1.25 (0.90,1.74)	0.183	3.02 (0.60,15.13)	0.180	2.33 (0.39,14.07)	0.356	0.27 (0.03,2.37)	0.235
		Normal, voiding dysfunction, not done	0.84 (0.46,1.55)	0.576	-	-	2.40 (0.18,31.61)	0.504	-	-
	Grade of operator	Consultant	Reference		Reference		Reference		Reference	
		Other	1.25 (0.92,1.69)	0.155	7.32 (1.35,39.63)	0.021	1.21 (0.08,17.77)	0.888	0.48 (0.08,2.78)	0.411
	Bladder injury		2.10 (1.23,3.58)	0.006	-		-	-	10.20 (1.56,66.76)	0.015

Other grade of operator = Associate specialist, subspec trainee, specialty trainee, other, staff grade, FTSTA

Characteristics studied did not significantly alter the outcomes for PUB. For AFS, the characteristics studied did not significantly alter PGII or SUI symptoms; however, there was a significantly increased odds of readmission to hospital with increased age (OR 2.03; 95% CI 1.10,3.77; *p* = 0.024). For colposuspension, increased age led to reduced odds of a positive outcome for change in SUI (OR 0.47; 95% CI 0.25,0.87; *p* = 0.017) and increased odds of bladder injury (OR 1.67; 95% CI 1.03,2.71; *p* = 0.036), return to hospital (OR 2.03; 95% CI 1.31,3.16; *p* = 0.002) and readmission (OR 1.82; 95% CI 1.12,2.95; *p* = 0.016).

### Secondary Analysis: Comparison Within Treatments: The Effect of Patient and Operative Characteristics: Secondary Population: Secondary Outcomes

Additionally, the secondary population of “all patients” was analysed, containing first and repeat surgeries. Repeat procedures led to reduced odds of success for PGII and SUI symptoms for RPT (OR 0.68; 95% CI 0.51,0.92; *p* = 0.012, OR 0.55; 95% CI 0.36,0.83; *p* = 0.004), PUB (OR 0.66; 95% CI 0.45,0.96; *p* = 0.031, OR 0.58; 95% CI 0.37,0.91; *p* = 0.018) and colposuspension (OR 0.43; 95% CI 0.22,0.86; *p* = 0.016, OR 0.23; 95% CI 0.08,0.66; *p* = 0.006). An increased odds of bladder injury (OR 1.67; 95% CI 1.10,2.53; *p* = 0.016) was seen with repeat RPT (appendix 3).

## Comment

### Principle Findings

High levels of success were seen following RPT, colposuspension and AFS for PGII and change in SUI symptoms, whereas outcomes after PUB were significantly poorer compared to RPT. The higher success rates were coupled with greater rates of complications. The more obstructive and surgically nuanced procedures of colposuspension and AFS were associated with more postoperative OAB. There was a significantly lower odds of return to hospital and readmission to hospital following PUB compared to RPT and a significantly higher odds of return and readmission to hospital following AFS.

With regard to patient characteristics, several features were apparent. Broadly speaking, the analysis suggested failure was associated with increased age, higher BMI, preoperative UDS with detrusor overactivity and bladder injury. Although a lower bladder injury rate was seen with higher BMI in RPT.

Bladder injury during RPT was more common with non-consultant grades and this may relate to these practitioners being on a learning curve; therefore, limiting the number of trainees performing these procedures and simulation models may help to address this [53].

The reduced success and increased bladder injury seen with repeat continence surgery may be due to scarring, nerve damage during periurethral dissection, or it may be that those patients had worse SUI with more severe neuromuscular compromise [54] or so therefore have a worse outcome regardless.

### Results in the Context of What is Known

While absolute success rates were found to be comparable to published literature [13, 43, 55–61], there are challenges with comparing existing papers due to the lack of a core outcome set with over 24 validated outcome measures for female incontinence [62]. Most literature is not high quality, with high or unclear risk of bias, particularly in older papers, which tended to be those including AFS and colposuspension, as they were less commonly performed in more recent years. This study provides an up-to-date overview of real life outcomes and complications.

### Clinical Implications

This study has shown that the large case numbers generated from the BSUG database can be utilised to produce meaningful results about success and complications which can be used to improve counselling and will be important for patients to consider when considering their options.

The primary multivariable analysis outcomes can assist patients to decide what treatment is more suitable to their wishes. If a “cure” of their SUI symptoms is sought, then an RPT, AFS or colposuspension is likely to be more appropriate; however, other patients may feel a lower success rate, coupled with a low complication rate, is more desirable.

The information regarding how patient characteristics affected outcomes in each treatment can be used to temper expectation in likely outcomes. The results from this analysis should not necessarily preclude patients with unfavourable characteristics from receiving interventions, as the success rates for these groups were still high and patient choice is important, but it must be reinforced that bespoke patient counselling is of vital importance to ensure patients are aware of the lower chance of success. With the knowledge that SUI and OAB occur more frequently in patients with higher BMI [2, 34] and outcomes are poorer, patients may wish to reduce this modifiable characteristic as surgery may be rendered unnecessary [2, 63].

Rates of return to theatre, return to hospital and readmission to hospital are particularly useful for patients counselling, and this has not previously been widely reported. Trials are less likely to report this type of information, whereas the BSUG database enables this. If a patient is at high risk of anaesthetic complications or lives rurally, an increased risk of return to theatre or return to hospital may be more impactful so this information can further aid decision-making.

### Research Implications

Now, more than ever, with the concern over mesh tapes, and the current limited options in some countries, it is important to be able to facilitate comparisons on a large scale such as this. National clinical databases are a valuable resource to assess trends, complications, and outcomes. Furthermore, clinical databases complement randomised controlled trials, providing real world clinical data to establish treatment effects and outcomes in varying clinical settings.

### Strengths and Limitations

This dataset is larger than others in the current literature and has been performed on a national level. Missing data is the main limitation as it is a non-mandatory database so patients may not have been entered onto it and also there are significant numbers of outcome data missing. For outcomes, there was no way to retrospectively gain more data as the cases were anonymised. The missing patient characteristics data was overcome using multiple imputation which requires complex statistical input. Missing data are substituted with an estimated value based on other relevant data. Multiple datasets are produced from multiple imputed values, and all datasets are analysed with the results being pooled to calculate the mean, variance, and confidence intervals. This maximally limits bias as it can be applied to cases where missing data is not random as well as those where data is missing at random.

The most relevant outcomes for SUI are subjective and therefore affected by the patient so can lead to bias. An objective measure of success for SUI would not align with the commonly accepted aim of treatment which is to improve quality of life. While there are many positives with patient reported outcomes, there are potential biases. There is a risk of recall bias where the patients’ memory is inaccurate, meaning an under or overestimation of the change in symptoms is reported. Patient Reported Outcomes Measures (PROM) should ideally be retrieved from the patient without contact with the operating team to reduce the risk of response bias which may occur if the patient feels influenced by the operating team. A regularly timed email or phone app could achieve this; however, resources were not enabled for this for BSUG at the time of data collection. An additional bias pertaining to PGII, relates to the preoperative expectations. If the clinical team has set low expectations, then any improvement will be very welcome; however, if the expectation is set very high and there is any deviation from this, then it may be poorly tolerated by the patient and their reported outcomes will reflect this.

The power of this database could be improved by auditing, making the database mandatory with longer term follow-up.

### Further Work

Databases like this can be used to develop a computerised algorithm involving a prediction model and decision making tool which allows patients to enter their characteristics and to estimate their chances of success and complications for all continence procedures to facilitate fully informed consent. It would help to establish which intervention may be preferred by patients based upon individual characteristics and understanding and acceptance of the risks involved. Some patients may prefer the highest chance of cure of SUI with the knowledge of a higher risk of complications compared to others who would accept lower chance of cure to avoid complications [64] leading to true personalised medicine.

The ideal database would have accurate complete data with validated information, avoiding bias, and requiring no additional efforts by clinicians or patients. The database would have a clear aim endorsed by all stakeholders and regularly report live relevant data while being responsive to the rapidly changing medical world [65]. An internationally agreed core outcome set for each condition should be considered to allow follow-up and international comparison of routine clinical care as developed by ICHOM for OAB [66].

## Conclusions

We strongly recommend that national bodies and international societies support the use of standardised, mandatory prospective data collection to enhance understanding of outcomes of surgical procedures.

## Supplementary Information

Below is the link to the electronic supplementary material.

Supplementary file1 (DOCX 26 KB)

Supplementary file2 (DOCX 54 KB)

Supplementary file3 (DOCX 229 KB)

## Data Availability

Application to view raw data is via the BSUG database committee.
