# Supplementary material for: Patient Reported Outcomes and Complications of Stress Incontinence Surgery: Effect of Patient Characteristics
Source: Int Urogynecol J. 2026 Jan 14;37(7):2081–94. doi: 10.1007/s00192-025-06507-1 (PMC13384967; doi:10.1007/s00192-025-06507-1)
Supplement: Supplementary file 1 — Supplementary file1 (DOCX 26 KB) [file 192_2025_6507_MOESM1_ESM.docx]

### Appendix 1:

Table 1: Baseline characteristics: Secondary population

| **Treatment** | **RPT** | **PUB** | **AFS** | **Colposuspension** |
| --- | --- | --- | --- | --- |
| N=31901 | n=24923 | n=4740 | n=538 | n=1700 |
| **Age (years), mean (SD)** | 52.54 (11.79) | 57.00 (15.64) | 51.90 (11.14) | 50.66 (11.09) |
| missing, n | 701 | 207 | 24 | 110 |
| **Age categories, n(%)** |  |  |  |  |
| Age less than 50 | 11236 (46.4%) | 1606 (35.4%) | 230 (44.7%) | 784 (49.3%) |
| Age between 50-79 | 12573 (51.9%) | 2527 (55.7%) | 280 (54.5%) | 789 (49.6%) |
| Age 80 and over | 413 (1.7%) | 400 (8.8%) | 4 (0.8%) | 17 (1.1%) |
| Missing, n | 701 | 207 | 24 | 110 |
|  |  |  |  |  |
| **BMI, mean (SD)** | 28.53 (4.89) | 29.25 (5.42) | 28.73 (4.81) | 28.63 (4.73) |
| Missing, n | 13330 | 3292 | 377 | 936 |
|  | | |  |  |
| **Pelvic floor exercises, n(%)** | |  |  |  |
| Yes | 19122 (87.6%) | 3349 (86.4%) | 371 (81.7%) | 1262 (83.9%) |
| No | 2716 (12.4%) | 527 (13.6%) | 83 (18.3%) | 243 (16.1%) |
| Missing, n | 3085 | 864 | 84 | 195 |
|  |  |  |  |  |
| **Grade of operator, n(%)** |  |  |  |  |
| Consultant | 19290 (78.7%) | 4012 (88.1%) | 409 (79.0%) | 1405 (84.1%) |
| Other | 5229 (21.3%) | 544 (11.9%) | 109 (21.1%) | 266 (15.9%) |
| Missing, n | 404 | 184 | 20 | 29 |
|  | | |  |  |
| **Pre-op urodynamic diagnosis, n(%)** | | |  |  |
| USI, USI+Void Dysfunction | 18111 (78.5%) | 2923 (73.6%) | 358 (77.5%) | 1278 (84.9%) |
| DOA, Mix, Mixed+void dys | 3731 (16.2%) | 738 (18.6%) | 85 (18.4%) | 171 (11.4%) |
| Normal, void dysf, not done | 1236 (5.4%) | 311 (7.8%) | 19 (4.1%) | 56 (3.7%) |
| Missing, n | 1845 | 768 | 76 | 195 |
|  | |  |  |  |
| **Incontinence Surgery Type, n(%)** | |  |  |  |
| Primary | 22742 (94.2%) | 3220 (73.5%) | 285 (57.1%) | 1248 (78.8%) |
| Repeat | 1411 (5.8%) | 1160 (26.5%) | 214 (42.9%) | 336 (21.2%) |
| Missing, n | 770 | 360 | 39 | 116 |

### Table 2: Patient reported outcomes: Secondary population: All patients

| **Treatment** | **RPT**  n=24923 | | **PUB**  n=4740 | | **AFS**  n=538 | | **Colposuspension** n=1700 | | **Total** |
| --- | --- | --- | --- | --- | --- | --- | --- | --- | --- |
| Population: All patients | | | | | | | | | |
| ***PGII, n(%)*** | | | | | | | | | |
| Better | 14495 | 90.5% | 1414 | 55.3% | 207 | 90.4% | 810 | 85.5% |  |
| Not better | 1527 | 9.5% | 1144 | 44.7% | 22 | 9.6% | 137 | 14.5% |  |
| Total | 16022 |  | 2558 |  | 229 |  | 947 |  | 19756 |
| *Missing PGII data* | *8901* |  | *2182* |  | *309* |  | *753* |  |  |
| ***SUI symptoms, n(%)*** | | | | | | | | | |
| Cured or Improved | 14231 | 96.4% | 1667 | 71.9% | 207 | 97.2% | 832 | 94.0% |  |
| No change or Worse | 529 | 3.6% | 653 | 28.1% | 6 | 2.8% | 53 | 6.0% |  |
| Total | 14760 |  | 2320 |  | 213 |  | 885 |  | 18,178 |
| *Missing SUI data* | *10163* |  | *2420* |  | *325* |  | *815* |  |  |
| **OAB** | | | | | | | | | |
| **OAB (Patients with pre-operative symptoms), n(%)** | | | | | | | | | |
| Cured or Improved | 5606 | 63.1% | 529 | 38.1% | 46 | 34.6% | 230 | 45.8% |  |
| No change or Worse | 3280 | 36.9% | 861 | 61.9% | 87 | 65.4% | 272 | 54.2% |  |
| Total | 8886 |  | 1390 |  | 133 |  | 502 |  | 10,911 |
| **OAB (Patients without pre-operative symptoms), n(%)** | | | | | | | | | |
| New symptoms | 542 | 9.6% | 22 | 3.0% | 9 | 13.0% | 49 | 13.8% |  |
| Never present | 5119 | 90.4% | 703 | 97.0% | 60 | 87.0% | 305 | 86.2% |  |
| Total | 5661 |  | 725 |  | 69 |  | 354 |  | 6,809 |
| *Missing OAB data* | *10376* |  | *2625* |  | *336* |  | *844* |  |  |
