# Supplementary material for: Patient Reported Outcomes and Complications of Stress Incontinence Surgery: Effect of Patient Characteristics
Source: Int Urogynecol J. 2026 Jan 14;37(7):2081–94. doi: 10.1007/s00192-025-06507-1 (PMC13384967; doi:10.1007/s00192-025-06507-1)
Supplement: Supplementary file 2 — Supplementary file2 (DOCX 54 KB) [file 192_2025_6507_MOESM2_ESM.docx]

# Appendix 2:

# Primary analysis of primary population

Table 1: PGII – Primary outcome

Table 2: Change in SUI

Table 3: Change in OAB with pre-op OAB

Table 4: Change in OAB without pre-op OAB

Table 5: Bladder injury

Table 6: Return to theatre

Table 7: Return to hospital

Table 8: Readmitted to hospital

Table 1: Univariable and multivariable analysis primary population: PGII

| **Characteristics** | **OR** | **95% CI** | **p value** |
| --- | --- | --- | --- |
| **Multivariable analysis (n=5122)** |  |  |  |
| **Treatment** |  |  |  |
| RPT | Reference |  |  |
| PUB | 0.13 | (0.09,0.17) | <0.001 |
| AFS | 1.12 | (0.25,4.93) | 0.882 |
| Colposuspension | 0.96 | (0.56,1.65) | 0.874 |
| **Age (decades)** | 0.89 | (0.83,0.95) | 0.001 |
| **BMI** | 0.95 | (0.93,0.97) | <0.001 |
| **Pelvic floor exercises** | 1.09 | (0.83,1.44) | 0.534 |
| **Pre operative urodynamic diagnosis** |  |  |  |
| USI, USI and voiding dysfunction | Reference |  |  |
| DOA, Mixed, mixed and voiding dysfunction | 0.62 | (0.50,0.77) | <0.001 |
| Normal, voiding dysfunction, not completed | 0.85 | (0.57,1.27) | 0.435 |
| **Grade of operator** |  |  |  |
| Consultant | Reference |  |  |
| Other (Associate specialist, subspec trainee, speciality trainee, other, staff grade, FTSTA) | 0.96 | (0.76,1.20) | 0.702 |
| **Bladder injury** | 0.61 | (0.38,0.98) | 0.042 |
|  |  |  |  |
| **Univariable analysis** |  |  |  |
| **Treatment (n=11825)** |  |  |  |
| RPT | Reference |  |  |
| PUB | 0.10 | (0.09,0.12) | <0.001 |
| AFS | 0.69 | (0.30,1.55) | 0.367 |
| Colposuspension | 0.67 | (0.48,0.92) | 0.014 |
| **Age (decades) (n=11606)** | 0.81 | (0.78,0.85) | <0.001 |
| **BMI (n=8072)** | 0.95 | (0.94,0.97) | <0.001 |
| **Pelvic floor exercises (n=10972)** | 1.16 | (0.96,1.40) | 0.113 |
| **Pre operative urodynamic diagnosis (n=11390)** |  |  |  |
| USI, USI and voiding dsyfunction | Reference |  |  |
| DOA, Mixed, mixed and voiding dysfunction | 0.61 | (0.52,0.70) | <0.001 |
| Normal, voiding dysfunction, not completed | 0.83 | (0.64,1.07) | 0.154 |
| **Grade of operator (n=11716)** |  |  |  |
| Consultant | Reference |  |  |
| Other (Associate specialist, subspec trainee, speciality trainee, other, staff grade, FTSTA) | 1.02 | (0.88,1.19) | 0.761 |
| **Bladder injury (n=11700)** | 1.06 | (0.75,1.50) | 0.735 |

Table 2: Univariable and multivariable analysis primary population: change in SUI

| **Characteristics** | **OR** | **95% CI** | **p value** |
| --- | --- | --- | --- |
| **Multivariable analysis (n=4993)** |  |  |  |
| **Treatment** |  |  |  |
| RPT | Reference |  |  |
| PUB | 0.10 | (0.06,0.14) | <0.001 |
| AFS | 0.26 | (0.06,1.17) | 0.079 |
| Colposuspension | 1.04 | (0.40,2.72) | 0.933 |
| **Age (decades)** | 0.81 | (0.73,0.90) | <0.001 |
| **BMI** | 0.96 | (0.94,0.99) | 0.005 |
| **Pelvic floor exercises** | 0.96 | (0.63,1.47) | 0.860 |
| **Pre operative urodynamic diagnosis** |  |  |  |
| USI, USI and voiding dysfunction | Reference |  |  |
| DOA, Mixed, mixed and voiding dysfunction | 0.78 | (0.56,1.09) | 0.140 |
| Normal, voiding dysfunction, not completed | 0.99 | (0.53,1.84) | 0.966 |
| **Grade of operator** |  |  |  |
| Consultant | Reference |  |  |
| Other (Associate specialist, subspec trainee, speciality trainee, other, staff grade, FTSTA) | 0.85 | (0.60,1.20) | 0.350 |
| **Bladder injury** | 0.57 | (0.27,1.20) | 0.138 |
|  |  |  |  |
| **Univariable analysis** |  |  |  |
| **Treatment (n=11072)** |  |  |  |
| RPT | Reference |  |  |
| PUB | 0.08 | (0.06,0.09) | <0.001 |
| AFS | 0.89 | (0.21,3.76) | 0.874 |
| Colposuspension | 0.95 | (0.53,1.71) | 0.859 |
| **Age (decades) (n=10953)** | 0.77 | (0.72,0.82) | <0.001 |
| **BMI (n=7865)** | 0.96 | (0.94,0.98) | <0.001 |
| **Pelvic floor exercises (n=10186)** | 1.26 | (0.96,1.65) | 0.092 |
| **Pre operative urodynamic diagnosis (n=10681)** |  |  |  |
| USI, USI and voiding dysfunction | Reference |  |  |
| DOA, Mixed, mixed and voiding dysfunction | 0.70 | (0.56,0.86) | 0.001 |
| Normal, voiding dysfunction, not completed | 0.78 | (0.54,1.14) | 0.200 |
| **Grade of operator (n=10970)** |  |  |  |
| Consultant | Reference |  |  |
| Other (Associate specialist, subspec trainee, speciality trainee, other, staff grade, FTSTA) | 0.99 | (0.80,1.24) | 0.961 |
| **Bladder injury (n=11030)** | 0.79 | (0.49,1.26) | 0.314 |

Table 3: Univariable and multivariable analysis primary population*:* change in OAB with pre-op OAB

| **Characteristics** | **OR** | **95% CI** | **p value** |
| --- | --- | --- | --- |
| **Multivariable analysis (n=2848)** |  |  |  |
| **Treatment** |  |  |  |
| RPT | Reference |  |  |
| PUB | 1.24 | (0.86,1.80) | 0.254 |
| AFS | 0.61 | (0.12,3.06) | 0.550 |
| Colposuspension | 0.99 | (0.60,1.65) | 0.970 |
| **Age (decades)** | 1.00 | (0.94,1.08) | 0.916 |
| **BMI** | 0.99 | (0.97,1.01) | 0.190 |
| **Pelvic floor exercises** | 0.89 | (0.67,1.18) | 0.407 |
| **Pre operative urodynamic diagnosis** |  |  |  |
| USI, USI and voiding dysfunction | Reference |  |  |
| DOA, Mixed, mixed and voiding dysfunction | 0.97 | (0.79,1.18) | 0.731 |
| Normal, voiding dysfunction, not completed | 1.17 | (0.76,1.79) | 0.485 |
| **Grade of operator** |  |  |  |
| Consultant | Reference |  |  |
| Other (Associate specialist, subspec trainee, speciality trainee, other, staff grade, FTSTA) | 0.99 | (0.80,1.22) | 0.896 |
| **Bladder injury** | 1.01 | (0.62,1.63) | 0.982 |
|  |  |  |  |
|  |  |  |  |
|  |  |  |  |
| **Univariable analysis** |  |  |  |
| **Treatment (n=6427)** |  |  |  |
| RPT | Reference |  |  |
| PUB | 0.59 | (0.48,0.73) | <0.001 |
| AFS | 0.46 | (0.21,1.00) | 0.051 |
| Colposuspension | 0.94 | (0.68,1.29) | 0.681 |
| **Age (decades) (n=6367)** | 0.97 | (0.93,1.01) | 0.148 |
| **BMI (n=4571)** | 0.99 | (0.97,1.00) | 0.074 |
| **Pelvic floor exercises (n=5948)** | 0.85 | (0.70,1.03) | 0.105 |
| **Pre operative urodynamic diagnosis (n=6121)** |  |  |  |
| USI, USI and voiding dsyfunction | Reference |  |  |
| DOA, Mixed, mixed and voiding dysfunction | 0.94 | (0.82,1.07) | 0.350 |
| Normal, voiding dysfunction, not completed | 0.97 | (0.72,1.30) | 0.819 |
| **Grade of operator (n=6360)** |  |  |  |
| Consultant | Reference |  |  |
| Other (Associate specialist, subspec trainee, speciality trainee, other, staff grade, FTSTA) | 0.94 | (0.81,1.08) | 0.383 |
| **Bladder injury (n=6396)** | 1.09 | (0.78,1.50) | 0.622 |

Table 4: Univariable and multivariable analysis primary population: change in OAB without pre-op OAB

| **Characteristics** | **OR** | **95% CI** | **p value** |
| --- | --- | --- | --- |
| **Multivariable analysis (n=2070)** |  |  |  |
| **Treatment** |  |  |  |
| RPT | Reference |  |  |
| PUB | 0.20 | (0.05,0.84) | 0.028 |
| AFS | 2.53 | (0.51,12.68) | 0.259 |
| Colposuspension | 1.11 | (0.46,2.66) | 0.822 |
| **Age (decades)** | 1.21 | (1.06,1.38) | 0.006 |
| **BMI** | 1.02 | (0.99,1.05) | 0.258 |
| **Pelvic floor exercises** | 0.94 | (0.56,1.58) | 0.815 |
| **Pre operative urodynamic diagnosis** |  |  |  |
| USI, USI and voiding dysfunction | Reference |  |  |
| DOA, Mixed, mixed and voiding dysfunction | 1.03 | (0.57,1.85) | 0.930 |
| Normal, voiding dysfunction, not completed | 0.94 | (0.52,1.68) | 0.824 |
| **Grade of operator** |  |  |  |
| Consultant | Reference |  |  |
| Other (Associate specialist, subspec trainee, speciality trainee, other, staff grade, FTSTA) | 1.46 | (1.02,2.10) | 0.041 |
| **Bladder injury** | 1.47 | (0.72,3.02) | 0.294 |
|  |  |  |  |
| **Univariable analysis** |  |  |  |
|  |  |  |  |
| **Characteristic** | **OR** | **95% CI** | **p value** |
| **Treatment (n=6427)** |  |  |  |
| RPT | Reference |  |  |
| PUB | 0.59 | (0.48,0.73) | <0.001 |
| AFS | 0.46 | (0.21,1.00) | 0.051 |
| Colposuspension | 0.94 | (0.68,1.29) | 0.681 |
| **Age (decades) (n=4325)** | 1.06 | (0.97,1.16) | 0.203 |
| **BMI (n=3269)** | 1.02 | (0.99,1.04) | 0.265 |
| **Pelvic floor exercises (n=4041)** | 0.91 | (0.63,1.33) | 0.641 |
| **Pre operative urodynamic diagnosis (n=4221)** |  |  |  |
| USI, USI and voiding dsyfunction | Reference |  |  |
| DOA, Mixed, mixed and voiding dysfunction | 1.12 | (0.73,1.72) | 0.591 |
| Normal, voiding dysfunction, not completed | 0.96 | (0.63,1.45) | 0.839 |
| **Grade of operator (n=4343)** |  |  |  |
| Consultant | Reference |  |  |
| Other (Associate specialist, subspec trainee, speciality trainee, other, staff grade, FTSTA) | 1.42 | (1.10,1.83) | 0.007 |
| **Bladder injury (n=4364)** | 1.12 | (0.63,1.99) | 0.705 |

Table 5: Univariable and multivariable analysis primary population: bladder injury

| **Characteristics** | **OR** | **95% CI** | **p value** |
| --- | --- | --- | --- |
| **Multivariable analysis (n=10251)** |  |  |  |
| **Treatment** |  |  |  |
| RPT | Reference |  |  |
| PUB | 0.03 | (0.00,0.19) | <0.001 |
| AFS | 1.45 | (0.50,4.25) | 0.496 |
| Colposuspension | 0.92 | (0.50,1.68) | 0.776 |
| **Age (decades)** | 1.10 | (1.00,1.22) | 0.060 |
| **BMI** | 0.93 | (0.91,0.96) | <0.001 |
| **Pre operative urodynamic diagnosis** |  |  |  |
| USI, USI and voiding dysfunction | Reference |  |  |
| DOA, Mixed, mixed and voiding dysfunction | 0.80 | (0.56,1.15) | 0.230 |
| Normal, voiding dysfunction, not completed | 0.55 | (0.29,1.04) | 0.065 |
| **Grade of operator** |  |  |  |
| Consultant | Reference |  |  |
| Other (Associate specialist, subspec trainee, speciality trainee, other, staff grade, FTSTA) | 3.72 | (2.88,4.80) | <0.001 |
|  |  |  |  |
| **Univariable analysis** |  |  |  |
| **Treatment (n=18883)** |  |  |  |
| RPT | Reference |  |  |
| PUB | 0.02 | (0.01,0.10) | <0.001 |
| AFS | 0.71 | (0.29,1.73) | 0.449 |
| Colposuspension | 0.80 | (0.51,1.27) | 0.346 |
| **Age (decades) (n=18472)** | 1.09 | (1.01,1.16) | 0.018 |
| **BMI (n=10906)** | 0.94 | (0.91,0.96) | <0.001 |
| **Pre operative urodynamic diagnosis (n=17752)** |  |  |  |
| USI, USI and voiding dsyfunction | Reference |  |  |
| DOA, Mixed, mixed and voiding dysfunction | 0.90 | (0.71,1.13) | 0.361 |
| Normal, voiding dysfunction, not completed | 0.58 | (0.37,0.93) | 0.024 |
| **Grade of operator (n=18683)** |  |  |  |
| Consultant | Reference |  |  |
| Other (Associate specialist, subspec trainee, speciality trainee, other, staff grade, FTSTA) | 3.63 | (3.02,4.36) | <0.001 |

Table 6: Univariable and multivariable analysis primary population: return to theatre

| **Characteristics** | **OR** | **95% CI** | **p value** |
| --- | --- | --- | --- |
| **Multivariable analysis** |  |  |  |
| **Treatment** |  |  |  |
| RPT | No return to theatre for PUB and AFS recorded | | |
| PUB |  |  |  |
| AFS |  |  |  |
| Colposuspension |  |  |  |
| **Age (decades)** | 0.00 | (0.00,0.00) | <0.001 |
| **BMI** | 0.00 | (0.00,0.00) | <0.001 |
| **Pre operative urodynamic diagnosis** |  |  |  |
| USI, USI and voiding dsyfunction | Reference |  |  |
| DOA, Mixed, mixed and voiding dysfunction | 0.00 | (0.00,0.00) | <0.001 |
| Normal, voiding dysfunction, not completed | 0.00 | (0.00,0.00) | <0.001 |
| **Grade of operator** |  |  |  |
| Consultant | Reference |  |  |
| Other (Associate specialist, subspec trainee, speciality trainee, other, staff grade, FTSTA) | 0.00 | (0.00,0.00) | <0.001 |
| **Bladder injury** | 0.00 | (0.00,0.00) | <0.001 |
|  |  |  |  |
| **Univariable analysis** |  |  |  |
| **Treatment** |  |  |  |
| RPT | No return to theatre for PUB and AFS recorded | | |
| PUB |  |  |  |
| AFS |  |  |  |
| Colposuspension |  |  |  |
| **Age (decades) (n=13205)** | 0.80 | (0.63,1.00) | 0.053 |
| **BMI (n=8818)** | 0.97 | (0.90,1.03) | 0.303 |
| **Pre operative urodynamic diagnosis (n=12953)** |  |  |  |
| USI, USI and voiding dsyfunction | Reference |  |  |
| DOA, Mixed, mixed and voiding dysfunction | 0.60 | (0.27,1.34) | 0.212 |
| Normal, voiding dysfunction, not completed | 1.29 | (0.45,3.69) | 0.630 |
| **Grade of operator (n=13336)** |  |  |  |
| Consultant | Reference |  |  |
| Other (Associate specialist, subspec trainee, speciality trainee, other, staff grade, FTSTA) | 3.88 | (2.26,6.66) | <0.001 |
| **Bladder injury (n=13325)** | 5.43 | (2.58,11.41) | <0.001 |

Table 7: Univariable and multivariable analysis primary population: return to hospital

| **Characteristics** | **OR** | **95% CI** | **p value** |
| --- | --- | --- | --- |
| **Multivariable analysis (n=4032)** |  |  |  |
| **Treatment** |  |  |  |
| RPT | Reference |  |  |
| PUB | 0.32 | (0.19,0.53) | <0.001 |
| AFS | 2.42 | (1.07,5.45) | 0.033 |
| Colposuspension | 1.05 | (0.62,1.78) | 0.848 |
| **Age (decades)** | 1.10 | (0.97,1.23) | 0.131 |
| **BMI** | 0.99 | (0.96,1.03) | 0.607 |
| **Pre operative urodynamic diagnosis** |  |  |  |
| USI, USI and voiding dsyfunction | Reference |  |  |
| DOA, Mixed, mixed and voiding dysfunction | 0.89 | (0.60,1.31) | 0.550 |
| Normal, voiding dysfunction, not completed | 1.41 | (0.80,2.49) | 0.240 |
| **Grade of operator** |  |  |  |
| Consultant | Reference |  |  |
| Other (Associate specialist, subspec trainee, speciality trainee, other, staff grade, FTSTA) | 1.27 | (0.88,1.82) | 0.199 |
| **Bladder injury** | 1.70 | (0.88,3.28) | 0.116 |
|  |  |  |  |
| **Characteristic** | **OR** | **95% CI** | **p value** |
| **Treatment (n=4981)** |  |  |  |
| RPT | Reference |  |  |
| PUB | 0.25 | (0.16,0.40) | <0.001 |
| AFS | 2.52 | (1.24,5.10) | 0.010 |
| Colposuspension | 1.01 | (0.64,1.59) | 0.964 |
| **Age (decades) (n=4943)** | 0.98 | (0.89,1.08) | 0.706 |
| **BMI (n=4263)** | 0.99 | (0.96,1.03) | 0.642 |
| **Pre operative urodynamic diagnosis (n=4733)** |  |  |  |
| USI, USI and voiding dsyfunction | Reference |  |  |
| DOA, Mixed, mixed and voiding dysfunction | 0.98 | (0.70,1.37) | 0.905 |
| Normal, voiding dysfunction, not completed | 1.43 | (0.89,2.30) | 0.136 |
| **Grade of operator (n=4933)** |  |  |  |
| Consultant | Reference |  |  |
| Other (Associate specialist, subspec trainee, speciality trainee, other, staff grade, FTSTA) | 1.27 | (0.93,1.74) | 0.131 |
| **Bladder injury (n=4959)** | 2.45 | (1.45,4.14) | 0.001 |

Table 8: Univariable and multivariable analysis primary population: readmitted to hospital

| **Characteristics** | **OR** | **95% CI** | **p value** |  |
| --- | --- | --- | --- | --- |
| **Multivariable analysis (n=8207)** |  |  |  |  |
| **Treatment** |  |  |  |  |
| RPT | Reference |  |  |  |
| PUB | 0.22 | (0.10,0.47) | <0.001 |  |
| AFS | 5.81 | (2.64,12.75) | <0.001 |  |
| Colposuspension | 1.56 | (0.93,2.61) | 0.092 |  |
| **Age (decades)** | 1.11 | (1.00,1.22) | 0.052 |  |
| **BMI** | 1.01 | (0.98,1.03) | 0.560 |  |
| **Pre operative urodynamic diagnosis** |  |  |  |  |
| USI, USI and voiding dsyfunction | Reference |  |  |  |
| DOA, Mixed, mixed and voiding dysfunction | 1.22 | (0.89,1.66) | 0.215 |  |
| Normal, voiding dysfunction, not completed | 0.80 | (0.44,1.44) | 0.455 |  |
| **Grade of operator** |  |  |  |  |
| Consultant | Reference |  |  |  |
| Other (Associate specialist, subspec trainee, speciality trainee, other, staff grade, FTSTA) | 1.27 | (0.95,1.69) | 0.106 |  |
| **Bladder injury** | 2.28 | (1.39,3.75) | 0.001 |  |
|  |  |  |  |  |
| **Univariable analysis** |  |  |  |  |
| **Treatment (n=13225)** |  |  |  |  |
| RPT | Reference |  |  |  |
| PUB | 0.20 | (0.11,0.39) | <0.001 |  |
| AFS | 4.84 | (2.43,9.65) | <0.001 |  |
| Colposuspension | 1.44 | (0.93,2.25) | 0.104 |  |
| **Age (decades)( n=12968)** | 1.01 | (0.93,1.09) | 0.815 |  |
| **BMI (n=8680)** | 1.01 | (0.99,1.04) | 0.358 |  |
| **Pre operative urodynamic diagnosis (n=12724)** |  |  |  |  |
| USI, USI and voiding dsyfunction | Reference |  |  |  |
| DOA, Mixed, mixed and voiding dysfunction | 1.05 | (0.81,1.35) | 0.724 |  |
| Normal, voiding dysfunction, not completed | 0.83 | (0.54,1.29) | 0.414 |  |
| **Grade of operator (n=13099)** |  |  |  |  |
| Consultant | Reference |  |  |  |
| Other (Associate specialist, subspec trainee, speciality trainee, other, staff grade, FTSTA) | 1.29 | (1.02,1.61) | 0.030 |  |
| **Bladder injury (n=13087)** | 2.22 | (1.49,3.29) | <0.001 |  |
