# Supplementary material for: Patient Reported Outcomes and Complications of Stress Incontinence Surgery: Effect of Patient Characteristics
Source: Int Urogynecol J. 2026 Jan 14;37(7):2081–94. doi: 10.1007/s00192-025-06507-1 (PMC13384967; doi:10.1007/s00192-025-06507-1)
Supplement: Supplementary file 3 — Supplementary file3 (DOCX 229 KB) [file 192_2025_6507_MOESM3_ESM.docx]

**Appendix 3: Statistical tables**

Table of Contents

[Primary population: Non-concomitant First surgery 4](#_Toc133160439)

[RPT 4](#_Toc133160440)

[RPT: PGII 4](#_Toc133160441)

[RPT: Change in SUI symptoms 5](#_Toc133160442)

[RPT: Change in OAB symptoms: Patients with pre-operative symptoms 6](#_Toc133160443)

[RPT: Change in OAB symptoms: Patients without pre-operative symptoms 7](#_Toc133160444)

[RPT: Bladder injury 8](#_Toc133160445)

[RPT: Return to theatre for procedure-related event within 72 hours 9](#_Toc133160446)

[RPT: Return to hospital within 30 days for procedure related event 10](#_Toc133160447)

[RPT: Readmitted to hospital within 30 days for procedure related event 11](#_Toc133160448)

[PUB 12](#_Toc133160449)

[PUB: PGII 12](#_Toc133160450)

[PUB: Change in SUI symptoms 13](#_Toc133160451)

[PUB: Change in OAB symptoms: Patients with pre-operative symptoms 14](#_Toc133160452)

[PUB: Change in OAB symptoms: Patients without pre-operative symptoms 15](#_Toc133160453)

[PUB: Bladder injury 16](#_Toc133160454)

[PUB: Return to theatre for procedure related event within 72 hours 17](#_Toc133160455)

[PUB: Return to hospital within 30 days for procedure related event 18](#_Toc133160456)

[PUB: Readmitted to hospital within 30 days for procedure related event 19](#_Toc133160457)

[AFS 20](#_Toc133160458)

[AFS: PGII 20](#_Toc133160459)

[AFS: Change in SUI symptoms 21](#_Toc133160460)

[AFS: Change in OAB symptoms: Patients with pre-operative symptoms 22](#_Toc133160461)

[AFS: Change in OAB symptoms: Patients without pre-operative symptoms 23](#_Toc133160462)

[AFS: Bladder injury 24](#_Toc133160463)

[AFS: Return to theatre for procedure-related event within 72 hours 24](#_Toc133160464)

[AFS: Return to hospital within 30 days for procedure related event 25](#_Toc133160465)

[AFS: Readmitted to hospital within 30 days for procedure related event 26](#_Toc133160466)

[Colposuspension 27](#_Toc133160467)

[Colposuspension: PGII 27](#_Toc133160468)

[Colposuspension: Change in SUI symptoms 28](#_Toc133160469)

[Colposuspension: Change in OAB symptoms: Patients with pre-operative symptoms 29](#_Toc133160470)

[Colposuspension: Change in OAB symptoms: Patients without pre-operative symptoms 30](#_Toc133160471)

[Colposuspension: Bladder injury 31](#_Toc133160472)

[Colposuspension: Return to theatre for procedure-related event within 72 hours 32](#_Toc133160473)

[Colposuspension: Return to hospital within 30 days for procedure related event 33](#_Toc133160474)

[Colposuspension: Readmitted to hospital within 30 days for procedure related event 34](#_Toc133160475)

[Secondary population: All patients 35](#_Toc133160476)

[RPT 35](#_Toc133160477)

[RPT: PGII 35](#_Toc133160478)

[RPT: Change in SUI symptoms 36](#_Toc133160479)

[RPT: Change in OAB symptoms: Patients with pre-operative symptoms 37](#_Toc133160480)

[RPT: Change in OAB symptoms: Patients without pre-operative symptoms 38](#_Toc133160481)

[RPT: Bladder injury 39](#_Toc133160482)

[RPT: Return to theatre for procedure-related event within 72 hours 40](#_Toc133160483)

[RPT: Return to hospital within 30 days for procedure related event 41](#_Toc133160484)

[*RPT:* Readmitted to hospital within 30 days for procedure related event 42](#_Toc133160485)

[PUB 43](#_Toc133160486)

[PUB: PGII 43](#_Toc133160487)

[PUB: Change in SUI symptoms 44](#_Toc133160488)

[*PUB :* Change in OAB symptoms: Patients with pre-operative symptoms 45](#_Toc133160489)

[*PUB:* Change in OAB symptoms: Patients without pre-operative symptoms 46](#_Toc133160490)

[PUB: Bladder injury 47](#_Toc133160491)

[*PUB:* Return to theatre for procedure-related event within 72 hours 48](#_Toc133160492)

[*PUB:* Return to hospital within 30 days for procedure related event 49](#_Toc133160493)

[*PUB:* Readmitted to hospital within 30 days for procedure related event 50](#_Toc133160494)

[AFS 51](#_Toc133160495)

[AFS: PGII 51](#_Toc133160496)

[*AFS:* Change of SUI symptoms 52](#_Toc133160497)

[*AFS:* Change in OAB symptoms: Patients with pre-operative symptoms 53](#_Toc133160498)

[AFS: Change in OAB symptoms: Patients without pre-operative symptoms 54](#_Toc133160499)

[*AFS:* Bladder injury 55](#_Toc133160500)

[*AFS: R*eturn to theatre for procedure-related event within 72 hours 56](#_Toc133160501)

[*AFS:* Return to hospital within 30 days for procedure related event 57](#_Toc133160502)

[*AFS:* Readmitted to hospital within 30 days for procedure related event 58](#_Toc133160503)

[Colposuspension 59](#_Toc133160504)

[Colposuspension: PGII 59](#_Toc133160505)

[Colposuspension: Change in SUI symptoms 60](#_Toc133160506)

[Colposuspension: Change in OAB symptoms: Patients with pre-operative symptoms 61](#_Toc133160507)

[Colposuspension: Change in OAB symptoms: Patients without pre-operative symptoms 62](#_Toc133160508)

[Colposuspension: Bladder injury 63](#_Toc133160509)

[Colposuspension: Return to theatre for procedure-related event within 72 hours 64](#_Toc133160510)

[Colposuspension: Return to theatre for procedure-related event within 72 hours 65](#_Toc133160511)

[Colposuspension: Readmitted to hospital within 30 days for procedure related event 66](#_Toc133160512)

# Primary population: Non-concomitant First surgery

# RPT

## RPT: PGII

| **RPT** | **Populations: Non-concomitant First surgery** | | |
| --- | --- | --- | --- |
| **Primary outcome:** |  | | |
| PGII |  | |  |
| No change vs Better |  | |  |
| (No change: A little better, No change, a little worse, much worse, very much worse |  | | |
| Better: Very much better, much better) |  | | |
| **n=5914** |  |  |  |
| **Multivariable analysis (accounting for Centre as a random effect)** | | | |
| Number of imputations = 66 |  |  |  |
| **Characteristics** | **Odds ratio** | **95% CI** | **p-value** |
| **Age (decades)** | 0.83 | (0.77,0.90) | <0.001 |
| **BMI** | 0.95 | (0.93,0.97) | <0.001 |
| **PFMT** | 1.11 | (0.84,1.47) | 0.472 |
| **Pre operative urodynamic diagnosis** |  |  |  |
| USI, USI and voiding dysfunction | Reference |  |  |
| DOA, Mixed, mixed and voiding dysfunction | 0.51 | (0.41,0.64) | <0.001 |
| Normal, voiding dysfunction, not completed | 0.92 | (0.60,1.43) | 0.718 |
| **Grade of operator** |  |  |  |
| Consultant | Reference |  |  |
| Other (Associate specialist, subspec trainee, speciality trainee, other, staff grade, FTSTA) | 0.85 | (0.68,1.06) | 0.146 |
| **Bladder injury** | 0.57 | (0.37,0.89) | 0.012 |

Table 1: Non-concomitant First surgery: RPT: PGII

## RPT: Change in SUI symptoms

| **RPT** | **Populations: Non-concomitant First surgery** | | |
| --- | --- | --- | --- |
| **Secondary outcome:** |  |  |  |
| Change of SUI |  |  |  |
| No change or worse vs Cured or improved |  |  |  |
| **n=5748** |  |  |  |
| **Multivariable analysis (accounting for Centre as a random effect)** | | | |
| Number of imputations = 66 |  |  |  |
| **Characteristics** | **Odds ratio** | **95% CI** | **p-value** |
| **Age (decades)** | 0.81 | (0.72,0.91) | <0.001 |
| **BMI** | 0.96 | (0.93,0.99) | 0.009 |
| **PFMT** | 1.04 | (0.66,1.62) | 0.866 |
| **Pre operative urodynamic diagnosis** |  |  |  |
| USI, USI and voiding dysfunction | Reference |  |  |
| DOA, Mixed, mixed and voiding dysfunction | 0.65 | (0.45,0.93) | 0.017 |
| Normal, voiding dysfunction, not completed | 0.96 | (0.47,1.94) | 0.900 |
| **Grade of operator** |  |  |  |
| Consultant | Reference |  |  |
| Other (Associate specialist, subspec trainee, speciality trainee, other, staff grade, FTSTA) | 0.72 | (0.51,1.03) | 0.069 |
| **Bladder injury** | 0.38 | (0.21,0.71) | 0.002 |

Table 2: Non-concomitant First surgery: RPT: SUI

## RPT: Change in OAB symptoms: Patients with pre-operative symptoms

| **RPT** | **Populations: Non-concomitant First surgery** | | |
| --- | --- | --- | --- |
|  |  |  |  |
| **Secondary outcome:** |  |  |  |
| Change in OAB symptoms: Patients with pre-operative symptoms | | | |
| No change or worse vs Cured or improved |  |  |  |
| **n=3340** |  |  |  |
| **Multivariable analysis (accounting for Centre as a random effect)** | | | |
| Number of imputations = 66 |  |  |  |
| **Characteristics** | **Odds ratio** | **95% CI** | **p-value** |
| **Age (decades)** | 0.98 | (0.92,1.04) | 0.511 |
| **BMI** | 0.99 | (0.97,1.00) | 0.092 |
| **PFMT** | 0.87 | (0.67,1.15) | 0.335 |
| **Pre operative urodynamic diagnosis** |  |  |  |
| USI, USI and voiding dysfunction | Reference |  |  |
| DOA, Mixed, mixed and voiding dysfunction | 0.92 | (0.76,1.11) | 0.386 |
| Normal, voiding dysfunction, not completed | 1.43 | (0.93,2.20) | 0.102 |
| **Grade of operator** |  |  |  |
| Consultant | Reference |  |  |
| Other (Associate specialist, subspec trainee, speciality trainee, other, staff grade, FTSTA) | 0.88 | (0.73,1.07) | 0.216 |
| **Bladder injury** | 0.92 | (0.60,1.41) | 0.696 |

Table 3: Non-concomitant First surgery: RPT: pre-op OAB

## RPT: Change in OAB symptoms: Patients without pre-operative symptoms

| **RPT** | **Populations: Non-concomitant First surgery** | | |
| --- | --- | --- | --- |
| **Secondary outcome:** |  |  |  |
| Change in OAB symptoms: Patients without pre-operative symptoms | | |  |
| Never present vs New symptoms |  |  |  |
| **n=2425** |  |  |  |
| **Multivariable analysis (accounting for Centre as a random effect)** | | | |
| Number of imputations = 66 |  |  |  |
| **Characteristics** | **Odds ratio** | **95% CI** | **p-value** |
| **Age (decades)** | 1.20 | (1.06,1.36) | 0.003 |
| **BMI** | 1.01 | (0.98,1.04) | 0.534 |
| **PFMT** | 1.01 | (0.62,1.65) | 0.975 |
| **Pre operative urodynamic diagnosis** |  |  |  |
| USI, USI and voiding dysfunction | Reference |  |  |
| DOA, Mixed, mixed and voiding dysfunction | 0.96 | (0.54,1.72) | 0.900 |
| Normal, voiding dysfunction, not completed | 0.86 | (0.49,1.53) | 0.615 |
| **Grade of operator** |  |  |  |
| Consultant | Reference |  |  |
| Other (Associate specialist, subspec trainee, speciality trainee, other, staff grade, FTSTA) | 1.50 | (1.07,2.09) | 0.018 |
| **Bladder injury** | 1.18 | (0.60,2.31) | 0.639 |

Table 4: Non-concomitant First surgery: RPT: no pre-op OAB

## RPT: Bladder injury

| **RPT** | **Populations: Non-concomitant First surgery** | | |
| --- | --- | --- | --- |
| **Secondary outcome:** |  |  |  |
| Bladder injury |  |  |  |
| Yes vs No |  |  |  |
| **n=8493** |  |  |  |
| **Multivariable analysis (accounting for Centre as a random effect)** | | | |
| Number of imputations = 66 |  |  |  |
| **Characteristics** | **Odds ratio** | **95% CI** | **p-value** |
| **Age (decades)** | 1.10 | (0.99,1.22) | 0.081 |
| **BMI** | 0.93 | (0.90,0.96) | <0.001 |
| **Pre operative urodynamic diagnosis** |  |  |  |
| USI, USI and voiding dysfunction | Reference |  |  |
| DOA, Mixed, mixed and voiding dysfunction | 0.75 | (0.51,1.10) | 0.140 |
| Normal, voiding dysfunction, not completed | 0.58 | (0.31,1.10) | 0.095 |
| **Grade of operator** |  |  |  |
| Consultant | Reference |  |  |
| Other (Associate specialist, subspec trainee, speciality trainee, other, staff grade, FTSTA) | 3.88 | (2.99,5.05) | <0.001 |

Table 5: Non-concomitant First surgery: RPT: Bladder injury

## RPT: Return to theatre for procedure-related event within 72 hours

| **RPT** | **Populations: Non-concomitant First surgery** | | |
| --- | --- | --- | --- |
| **Secondary outcome:** |  |  |  |
| Return to theatre for procedure-related event within 72 hours | |  |  |
| Yes vs No |  |  |  |
| **n=6,941** |  |  |  |
| **Multivariable analysis (accounting for Centre as a random effect)** | | | |
| Number of imputations = 66 |  |  |  |
| **Characteristics** | **Odds ratio** | **95% CI** | **p-value** |
| **Age (decades)** | 0.90 | (0.68,1.19) | 0.461 |
| **BMI** | 0.98 | (0.91,1.05) | 0.530 |
| **Pre operative urodynamic diagnosis** |  |  |  |
| USI, USI and voiding dysfunction | Reference |  |  |
| DOA, Mixed, mixed and voiding dysfunction | 0.48 | (0.17,1.42) | 0.185 |
| Normal, voiding dysfunction, not completed | 1.45 | (0.42,5.09) | 0.558 |
| **Grade of operator** |  |  |  |
| Consultant | Reference |  |  |
| Other (Associate specialist, subspec trainee, speciality trainee, other, staff grade, FTSTA) | 3.81 | (1.96,7.41) | <0.001 |
| **Bladder injury** | 4.23 | (1.81,9.90) | 0.001 |

Table 6: Non-concomitant First surgery: RPT: return to theatre

## RPT: Return to hospital within 30 days for procedure related event

| **RPT** | **Populations: Non-concomitant First surgery** | | |
| --- | --- | --- | --- |
| **Secondary outcome:** |  |  |  |
| Return to hospital within 30 days for procedure related event | |  |  |
| Yes vs No |  |  |  |
| **n=2,814** |  |  |  |
| **Multivariable analysis (accounting for Centre as a random effect)** | | | |
| Number of imputations = 66 |  |  |  |
| **Characteristics** | **Odds ratio** | **95% CI** | **p-value** |
| **Age (decades)** | 1.00 | (0.86,1.15) | 0.956 |
| **BMI** | 0.99 | (0.95,1.03) | 0.594 |
| **Pre operative urodynamic diagnosis** |  |  |  |
| USI, USI and voiding dysfunction | Reference |  |  |
| DOA, Mixed, mixed and voiding dysfunction | 0.85 | (0.54,1.33) | 0.469 |
| Normal, voiding dysfunction, not completed | 1.34 | (0.70,2.57) | 0.378 |
| **Grade of operator** |  |  |  |
| Consultant | Reference |  |  |
| Other (Associate specialist, subspec trainee, speciality trainee, other, staff grade, FTSTA) | 1.25 | (0.84,1.86) | 0.270 |
| **Bladder injury** | 1.70 | (0.85,3.42) | 0.134 |

Table 7: Non-concomitant First surgery: RPT: return to hospital

## RPT: Readmitted to hospital within 30 days for procedure related event

| **RPT** | **Populations: Non-concomitant First surgery** | | |
| --- | --- | --- | --- |
| **Secondary outcome:** |  |  |  |
| Readmitted to hospital within 30 days for procedure related event | |  |  |
| Yes vs No |  |  |  |
| **n=6,828** |  |  |  |
| **Multivariable analysis (accounting for Centre as a random effect)** | | | |
| Number of imputations = 66 |  |  |  |
| **Characteristics** | **Odds ratio** | **95% CI** | **p-value** |
| **Age (decades)** | 1.05 | (0.94,1.17) | 0.357 |
| **BMI** | 1.00 | (0.98,1.03) | 0.794 |
| **Pre operative urodynamic diagnosis** |  |  |  |
| USI, USI and voiding dysfunction | Reference |  |  |
| DOA, Mixed, mixed and voiding dysfunction | 1.25 | (0.90,1.74) | 0.183 |
| Normal, voiding dysfunction, not completed | 0.84 | (0.46,1.55) | 0.576 |
| **Grade of operator** |  |  |  |
| Consultant | Reference |  |  |
| Other (Associate specialist, subspec trainee, speciality trainee, other, staff grade, FTSTA) | 1.25 | (0.92,1.69) | 0.155 |
| **Bladder injury** | 2.10 | (1.23,3.58) | 0.006 |

Table 8: Non-concomitant First surgery: RPT: readmitted to hospital

# PUB

## PUB: PGII

| **PUB** | **Populations:** Non-concomitant First surgery | | |
| --- | --- | --- | --- |
| **Primary outcome:** |  | | |
| PGII |  | | |
| No change vs Better |  | | |
| (No change: A little better, No change, a little worse, much worse, very much worse | | | |
| Better: Very much better, much better) | | | |
| **n=861** |  |  |  |
| **Multivariable analysis (accounting for Centre as a random effect)** | | | |
| Number of imputations = 66 |  |  |  |
| **Characteristics** | **Odds ratio** | **95% CI** | **p-value** |
| **Age (decades)** | 1.04 | (0.95,1.14) | 0.425 |
| **BMI** | 0.98 | (0.95,1.02) | 0.341 |
| **PFMT** | 1.04 | (0.63,1.72) | 0.880 |
| **Pre operative urodynamic diagnosis** |  |  |  |
| USI, USI and voiding dysfunction | Reference |  |  |
| DOA, Mixed, mixed and voiding dysfunction | 1.11 | (0.78,1.58) | 0.572 |
| Normal, voiding dysfunction, not completed | 0.51 | (0.26,0.99) | 0.047 |
| **Grade of operator** |  |  |  |
| Consultant | Reference |  |  |
| Other (Associate specialist, subspec trainee, speciality trainee, other, staff grade, FTSTA) | 0.71 | (0.46,1.09) | 0.117 |
| **Bladder injury** | Omitted |  |  |

Table 9:Non-concomitant First surgery: PUB: PGII

## PUB: Change in SUI symptoms

| **PUB** | **Populations:** Non-concomitant First surgery | | |
| --- | --- | --- | --- |
| **Secondary outcome:** |  |  |  |
| Change of SUI |  |  |  |
| No change or worse vs Cured or improved |  |  |  |
| **n= 838** |  |  |  |
| **Multivariable analysis (accounting for Centre as a random effect)** | | | |
| Number of imputations = 66 |  |  |  |
| **Characteristics** | **Odds ratio** | **95% CI** | **p-value** |
| **Age (decades)** | 0.93 | (0.83,1.04) | 0.180 |
| **BMI** | 0.99 | (0.95,1.03) | 0.468 |
| **PFMT** | 1.17 | (0.66,2.07) | 0.599 |
| **Pre operative urodynamic diagnosis** |  |  |  |
| USI, USI and voiding dysfunction | Reference |  |  |
| DOA, Mixed, mixed and voiding dysfunction | 1.02 | (0.67,1.56) | 0.910 |
| Normal, voiding dysfunction, not completed | 0.59 | (0.29,1.22) | 0.156 |
| **Grade of operator** |  |  |  |
| Consultant | Reference |  |  |
| Other (Associate specialist, subspec trainee, speciality trainee, other, staff grade, FTSTA) | 0.96 | (0.57,1.63) | 0.890 |
| **Bladder injury** | Omitted |  |  |

Table 10:Non-concomitant First surgery: PUB:SUI

## PUB: Change in OAB symptoms: Patients with pre-operative symptoms

| **PUB** | **Populations:** Non-concomitant First surgery | | |
| --- | --- | --- | --- |
| **Secondary outcome:** |  |  |  |
| Change in OAB symptoms: Patients with pre-operative symptoms | | |  |
| No change or worse vs Cured or improved |  |  |  |
| **n=505** |  |  |  |
| **Multivariable analysis (accounting for Centre as a random effect)** | | | |
| Number of imputations = 66 |  |  |  |
| **Characteristics** | **Odds ratio** | **95% CI** | **p-value** |
| **Age (decades)** | 1.01 | (0.89,1.15) | 0.846 |
| **BMI** | 1.01 | (0.97,1.06) | 0.665 |
| **PFMT** | 0.98 | (0.51,1.90) | 0.955 |
| **Pre operative urodynamic diagnosis** |  |  |  |
| USI, USI and voiding dysfunction | Reference |  |  |
| DOA, Mixed, mixed and voiding dysfunction | 1.37 | (0.89,2.11) | 0.157 |
| Normal, voiding dysfunction, not completed | 0.91 | (0.33,2.48) | 0.851 |
| **Grade of operator** |  |  |  |
| Consultant | Reference |  |  |
| Other (Associate specialist, subspec trainee, speciality trainee, other, staff grade, FTSTA) | 0.58 | (0.30,1.13) | 0.109 |
| **Bladder injury** | Omitted |  |  |

Table 11:Non-concomitant First surgery: PUB: pre-OAB

## PUB: Change in OAB symptoms: Patients without pre-operative symptoms

| **PUB** | **Populations:** Non-concomitant First surgery | | | |
| --- | --- | --- | --- | --- |
| **Secondary outcome:** |  |  | |  |
| Change in OAB symptoms: Patients without pre-operative symptoms | | | |  |
| Never present vs New symptoms |  |  | |  |
| **n=319** |  |  | |  |
| **Multivariable analysis (accounting for Centre as a random effect)** | | | | |
| Number of imputations = 4 |  | |  |  |
| **Characteristics** | **Odds ratio** | | **95% CI** | **p-value** |
| **Age (decades)** | 1.19 | | (0.69,2.04) | 0.528 |
| **BMI** | 1.11 | | (0.94,1.30) | 0.219 |
| **PFMT** | 331237.20 | | #VALUE! Unable to calculate a CI | 0.988 |
| **Pre operative urodynamic diagnosis** |  | |  |  |
| USI, USI and voiding dysfunction | Reference | |  |  |
| DOA, Mixed, mixed and voiding dysfunction | 0.00 | | #VALUE! Unable to calculate a CI | 0.994 |
| Normal, voiding dysfunction, not completed | 0.00 | | #VALUE! Unable to calculate a CI | 0.996 |
| **Grade of operator** |  | |  |  |
| Consultant | Reference | |  |  |
| Other (Associate specialist, subspec trainee, speciality trainee, other, staff grade, FTSTA) | 1.52 | | (0.17,13.81) | 0.710 |
| **Bladder injury** | Omitted | |  |  |

Table 12:Non-concomitant First surgery: PUB: no pre-op OAB

## PUB: Bladder injury

| **PUB** | **Populations:** Non-concomitant First surgery | | |
| --- | --- | --- | --- |
| **Secondary outcome:** |  |  |  |
| Bladder injury |  | ERROR |  |
| Yes vs No |  |  |  |
| **n=** |  |  |  |
| **Multivariable analysis (accounting for Centre as a random effect)** | | | |
| Number of imputations |  |  |  |
| **Characteristics** | **Odds ratio** | **95% CI** | **p-value** |
| **Age (decades)** |  |  |  |
| **BMI** |  |  |  |
| **Pre operative urodynamic diagnosis** |  |  |  |
| USI, USI and voiding dysfunction |  |  |  |
| DOA, Mixed, mixed and voiding dysfunction |  |  |  |
| Normal, voiding dysfunction, not completed |  |  |  |
| **Grade of operator** |  |  |  |
| Consultant |  |  |  |
| Other (Associate specialist, subspec trainee, speciality trainee, other, staff grade, FTSTA) |  |  |  |
| **Surgery type** |  |  |  |
| First |  |  |  |
| Repeat |  |  |  |

Table 13:Non-concomitant First surgery: PUB: Bladder injury

## PUB: Return to theatre for procedure related event within 72 hours

| **PUB** | | **Populations:** Non-concomitant First surgery | | |
| --- | --- | --- | --- | --- |
| **Secondary outcome:** | |  |  |  |
| Return to theatre for procedure-related event within 72 hours | | | ERROR |  |
| Yes vs No | |  |  |  |
| **n=** | |  |  |  |
| **Multivariable analysis (accounting for Centre as a random effect)** | | | | |
| Number of imputations |  | |  |  |
| **Characteristics** | **Odds ratio** | | **95% CI** | **p-value** |
| **Age (decades)** |  | |  |  |
| **BMI** |  | |  |  |
| **Pre operative urodynamic diagnosis** |  | |  |  |
| USI, USI and voiding dysfunction |  | |  |  |
| DOA, Mixed, mixed and voiding dysfunction |  | |  |  |
| Normal, voiding dysfunction, not completed |  | |  |  |
| **Grade of operator** |  | |  |  |
| Consultant |  | |  |  |
| Other (Associate specialist, subspec trainee, speciality trainee, other, staff grade, FTSTA) |  | |  |  |
| **Bladder injury** |  | |  |  |
| **Surgery type** |  | |  |  |
| First |  | |  |  |
| Repeat |  | |  |  |

Table 14:Non-concomitant First surgery: PUB: return to theatre

## PUB: Return to hospital within 30 days for procedure related event

| **PUB** | **Populations:** Non-concomitant First surgery | | |
| --- | --- | --- | --- |
| **Secondary outcome:** |  |  |  |
| Return to hospital within 30 days for procedure related event | |  |  |
| Yes vs No |  |  |  |
| **n=862** |  |  |  |
| **Multivariable analysis (accounting for Centre as a random effect)** | | | |
| Number of imputations = 66 |  |  |  |
| **Characteristics** | **Odds ratio** | **95% CI** | **p-value** |
| **Age (decades)** | 1.26 | (0.91,1.75) | 0.168 |
| **BMI** | 1.04 | (0.92,1.17) | 0.513 |
| **Pre operative urodynamic diagnosis** |  |  |  |
| USI, USI and voiding dysfunction | Reference |  |  |
| DOA, Mixed, mixed and voiding dysfunction | 2.63 | (0.82,8.49) | 0.106 |
| Normal, voiding dysfunction, not completed | 4.02 | (1.02,15.90) | 0.047 |
| **Grade of operator** |  |  |  |
| Consultant | Reference |  |  |
| Other (Associate specialist, subspec trainee, speciality trainee, other, staff grade, FTSTA) | 3.87 | (0.90,16.58) | 0.068 |
| **Bladder injury** | Omitted |  |  |

Table 15:Non-concomitant First surgery: PUB: return to hospital

## PUB: Readmitted to hospital within 30 days for procedure related event

| **PUB** | **Populations:** Non-concomitant First surgery | | |
| --- | --- | --- | --- |
| **Secondary outcome:** |  |  |  |
| Readmitted to hospital within 30 days for procedure related event | |  |  |
| Yes vs No |  |  |  |
| **n=961** |  |  |  |
| **Multivariable analysis (accounting for Centre as a random effect)** | | | |
| Number of imputations = 66 |  |  |  |
| **Characteristics** | **Odds ratio** | **95% CI** | **p-value** |
| **Age (decades)** | 1.10 | (0.65,1.87) | 0.723 |
| **BMI** | 0.96 | (0.77,1.19) | 0.710 |
| **Pre operative urodynamic diagnosis** |  |  |  |
| USI, USI and voiding dysfunction | Reference |  |  |
| DOA, Mixed, mixed and voiding dysfunction | 3.02 | (0.60,15.13) | 0.180 |
| Normal, voiding dysfunction, not completed | 0.00 | #VALUE! Unable to calculate CI | 0.995 |
| **Grade of operator** |  |  |  |
| Consultant | Reference |  |  |
| Other (Associate specialist, subspec trainee, speciality trainee, other, staff grade, FTSTA) | 7.32 | (1.35,39.63) | 0.021 |
| **Bladder injury** | Omitted |  |  |

Table 16:Non-concomitant First surgery: PUB: readmitted to hospital

# AFS

## AFS: PGII

| **AFS** | **Populations:** Non concomitant First surgery | | | | |
| --- | --- | --- | --- | --- | --- |
| **Primary outcome:** |  | | | | |
| PGII |  | | |  | |
| No change vs Better |  | | |  | |
| (No change: A little better, No change, a little worse, much worse, very much worse | | | | | |
| Better: Very much better, much better) | |  | | | |
| **n=48** | |  |  | |  |
| **Multivariable analysis (accounting for Centre as a random effect)** | |  |  | |  |
| Number of imputations = 31 | |  |  | |  |
| **Characteristics** | | **Odds ratio** | **95% CI** | | **p-value** |
| **Age (decades)** | | 0.47 | (0.10,2.32) | | 0.354 |
| **BMI** | | 0.66 | (0.24,1.87) | | 0.435 |
| **PFMT** | | 0.00 | #VALUE! Unable to calculate CI | | 0.997 |
| **Pre operative urodynamic diagnosis** | |  |  | |  |
| USI, USI and voiding dysfunction | | Reference |  | |  |
| DOA, Mixed, mixed and voiding dysfunction | | 1.23 | (0.03,52.24) | | 0.914 |
| Normal, voiding dysfunction, not completed | | 0.09 | (0.00,20.21) | | 0.388 |
| **Grade of operator** | |  |  | |  |
| Consultant | | Reference |  | |  |
| Other (Associate specialist, subspec trainee, speciality trainee, other, staff grade, FTSTA) | | 0.01 | (0.00,551.52) | | 0.410 |
| **Bladder injury** | | 45400000.00 | #VALUE! Unable to calculate CI | | 1.000 |

Table 17:Non-concomitant First surgery: AFS: PGII

## AFS: Change in SUI symptoms

| **AFS** | **Populations:** Non concomitant First surgery | | | |
| --- | --- | --- | --- | --- |
| **Secondary outcome:** |  |  |  | |
| Change of SUI |  |  |  | |
| No change or worse vs Cured or improved |  |  |  | |
| **n=45** |  |  |  | |
| **Multivariable analysis (accounting for Centre as a random effect)** | | | | |
| Number of imputations = 3 |  |  | | 3 |
| **Characteristics** | **Odds ratio** | **95% CI** | | **p-value** |
| **Age (decades)** | 2.97 | (0.04,214.36) | | 0.615 |
| **BMI** | 0.60 | (0.19,1.92) | | 0.380 |
| **PFMT** | 0.00 | #VALUE! Unable to calculate CI | | 0.998 |
| **Pre operative urodynamic diagnosis** |  |  | |  |
| USI, USI and voiding dysfunction | Reference |  | |  |
| DOA, Mixed, mixed and voiding dysfunction | 3780000 000.00 | #VALUE! Unable to calculate CI | | 0.999 |
| Normal, voiding dysfunction, not completed | 0.07 | (0.00,2041.87) | | 0.616 |
| **Grade of operator** |  |  | |  |
| Consultant | Reference |  | |  |
| Other (Associate specialist, subspec trainee, speciality trainee, other, staff grade, FTSTA) | 0.00 | (0.00,366.06) | | 0.287 |
| **Bladder injury** | 973000000.00 | #VALUE! Unable to calculate CI | | 0.999 |

Table 18:Non-concomitant First surgery: AFS: SUI

## AFS: Change in OAB symptoms: Patients with pre-operative symptoms

| **AFS** | **Populations:** Non concomitant First surgery | | |
| --- | --- | --- | --- |
| **Secondary outcome:** |  |  |  |
| Change in OAB symptoms: Patients with pre-operative symptoms | | | |
| No change or worse vs Cured or improved |  |  |  |
| **n=23** |  |  |  |
| **Multivariable analysis (accounting for Centre as a random effect)** | | |  |
| Number of imputations = 19 |  |  |  |
| **Characteristics** | **Odds ratio** | **95% CI** | **p-value** |
| **Age (decades)** | 4.93 | (0.02,1114.89) | 0.564 |
| **BMI** | 1.19 | (0.37,3.84) | 0.766 |
| **PFMT** | 0.00 | #VALUE! Unable to calculate CI | 0.993 |
| **Pre operative urodynamic diagnosis** |  |  |  |
| USI, USI and voiding dysfunction | Reference |  |  |
| DOA, Mixed, mixed and voiding dysfunction | 0.05 | (0.00,7689.26) | 0.617 |
| Normal, voiding dysfunction, not completed | 0.00 | #VALUE! Unable to calculate CI | 0.996 |
| **Grade of operator** |  |  |  |
| Consultant | Reference |  |  |
| Other (Associate specialist, subspec trainee, speciality trainee, other, staff grade, FTSTA) | 5660000000000000000.00 | #VALUE! Unable to calculate CI | 0.999 |
| **Bladder injury** | Omitted |  |  |

Table 19:Non-concomitant First surgery: AFS: Pre-op OAB

## AFS: Change in OAB symptoms: Patients without pre-operative symptoms

| **AFS** | **Populations:** Non concomitant First surgery | | |
| --- | --- | --- | --- |
| **Secondary outcome:** |  |  |  |
| Change in OAB symptoms: Patients without pre-operative symptoms | | |  |
| Never present vs New symptoms |  |  |  |
| **n=21** |  |  |  |
| **Multivariable analysis (accounting for Centre as a random effect)** | | |  |
| Number of imputations = 4 |  |  |  |
| **Characteristics** | **Odds ratio** | **95% CI** | **p-value** |
| **Age (decades)** | 1.14 | (0.08,17.08) | 0.916 |
| **BMI** | 0.99 | (0.75,1.32) | 0.965 |
| **PFMT** | 387048.30 | #VALUE! Unable to calculate CI | 0.996 |
| **Pre operative urodynamic diagnosis** |  |  |  |
| USI, USI and voiding dysfunction | Reference |  |  |
| DOA, Mixed, mixed and voiding dysfunction | 8.27 | (0.28,247.51) | 0.223 |
| Normal, voiding dysfunction, not completed | Omitted |  |  |
| **Grade of operator** |  |  |  |
| Consultant | Reference |  |  |
| Other (Associate specialist, subspec trainee, speciality trainee, other, staff grade, FTSTA) | 0.00 | #VALUE! Unable to calculate CI | 0.997 |
| **Bladder injury** | 0.28 | #VALUE! Unable to calculate CI | 1.000 |

Table 20:Non-concomitant First surgery: AFS: no pre-op OAB

## AFS: Bladder injury

| **AFS** | **Populations:** Non concomitant First surgery | | | |
| --- | --- | --- | --- | --- |
| **Secondary outcome:** |  |  |  |  |
| Bladder injury |  |  |  |  |
| Yes vs No |  |  |  |  |
| **n=91** |  |  |  |  |
| **Multivariable analysis (accounting for Centre as a random effect)** | | |  |  |
| Number of imputations = 29 |  |  |  |  |
| **Characteristics** | **Odds ratio** | **95% CI** | **p-value** |  |
| **Age (decades)** | 0.08 | (0.00,4.09) | 0.208 |  |
| **BMI** | 0.88 | (0.43,1.80) | 0.729 |  |
| **Pre operative urodynamic diagnosis** |  |  |  |  |
| USI, USI and voiding dysfunction | Reference |  |  |  |
| DOA, Mixed, mixed and voiding dysfunction | 0.00 | #VALUE! Unable to calculate CI | 0.996 |  |
| Normal, voiding dysfunction, not completed | 0.00 | #VALUE! Unable to calculate CI | 0.996 |  |
| **Grade of operator** |  |  |  |  |
| Consultant | Reference |  |  |  |
| Other (Associate specialist, subspec trainee, speciality trainee, other, staff grade, FTSTA) | 0.36 | (0.00,1171.97) | 0.802 |  |

Table 21:Non-concomitant First surgery: AFS: bladder injury

## AFS: Return to theatre for procedure-related event within 72 hours

| **AFS** | **Populations:** Non concomitant First surgery | | |
| --- | --- | --- | --- |
| **Secondary outcome:** |  |  |  |
| Return to theatre for procedure-related event within 72 hours | |  |  |
| Yes vs No | **All no for the outcome** | | |
| **n=** |  |  |  |

Table 22:Non-concomitant First surgery: AFS: return to theatre

## AFS: Return to hospital within 30 days for procedure related event

| **AFS** | **Population:** Non concomitant First surgery | | |
| --- | --- | --- | --- |
| **Secondary outcome:** |  |  |  |
| Return to hospital within 30 days for procedure related event | |  |  |
| Yes vs No |  |  |  |
| **n=53** |  |  |  |
| **Multivariable analysis (accounting for Centre as a random effect)** | | |  |
| Number of imputations = 2 |  |  |  |
| **Characteristics** | **Odds ratio** | **95% CI** | **p-value** |
| **Age (decades)** | 0.95 | (0.53,1.71) | 0.867 |
| **BMI** | 0.85 | (0.68,1.07) | 0.162 |
| **Pre operative urodynamic diagnosis** |  |  |  |
| USI, USI and voiding dysfunction | Reference |  |  |
| DOA, Mixed, mixed and voiding dysfunction | 0.41 | (0.04,3.96) | 0.444 |
| Normal, voiding dysfunction, not completed | 0.00 | #VALUE! Unable to calculate CI | 0.990 |
| **Grade of operator** |  |  |  |
| Consultant | Reference |  |  |
| Other (Associate specialist, subspec trainee, speciality trainee, other, staff grade, FTSTA) | 0.00 | #VALUE! Unable to calculate CI | 0.995 |
| **Bladder injury** | 0.16 | #VALUE! Unable to calculate CI | 1.000 |

Table 23:Non-concomitant First surgery: AFS: return to hospital

## AFS: Readmitted to hospital within 30 days for procedure related event

| **AFS** | **Populations:** Non concomitant First surgery | | |
| --- | --- | --- | --- |
| **Secondary outcome:** |  |  |  |
| Readmitted to hospital within 30 days for procedure related event | |  |  |
| Yes vs No |  |  |  |
| **n=58** |  |  |  |
| **Multivariable analysis (accounting for Centre as a random effect)** | | |  |
| Number of imputations = 63 |  |  |  |
| **Characteristics** | **Odds ratio** | **95% CI** | **p-value** |
| **Age (decades)** | 2.03 | (1.10,3.77) | 0.024 |
| **BMI** | 1.07 | (0.85,1.34) | 0.572 |
| **Pre operative urodynamic diagnosis** |  |  |  |
| USI, USI and voiding dysfunction | Reference |  |  |
| DOA, Mixed, mixed and voiding dysfunction | 2.33 | (0.39,14.07) | 0.356 |
| Normal, voiding dysfunction, not completed | 2.40 | (0.18,31.61) | 0.504 |
| **Grade of operator** |  |  |  |
| Consultant | Reference |  |  |
| Other (Associate specialist, subspec trainee, speciality trainee, other, staff grade, FTSTA) | 1.21 | (0.08,17.77) | 0.888 |
| **Bladder injury** | 0.00 | #VALUE! Unable to calculate CI | 0.996 |

Table 24:Non-concomitant First surgery: AFS: readmitted to hospital

# Colposuspension

## Colposuspension: PGII

| **Colposuspension** | **Populations:**  Non concomitant First surgery patients | | |
| --- | --- | --- | --- |
| **Primary outcome:** |  |  | |
| PGII |  |  | |
| No change vs Better |  |  | |
| (No change: A little better, No change, a little worse, much worse, very much worse | | | |
| Better: Very much better, much better) |  | | |
| **n=330** |  |  | |
| **Multivariable analysis (accounting for Centre as a random effect)** | | | |
| Number of imputations = 66 |  |  |  |
| **Characteristics** | **Odds ratio** | **95% CI** | **p-value** |
| **Age (decades)** | 0.82 | (0.59,1.12) | 0.215 |
| **BMI** | 0.98 | (0.89,1.08) | 0.714 |
| **PFMT** | 1.00 | (0.34,2.93) | 0.999 |
| **Pre operative urodynamic diagnosis** |  |  |  |
| USI, USI and voiding dysfunction | Reference |  |  |
| DOA, Mixed, mixed and voiding dysfunction | 2.81 | (0.81,9.69) | 0.102 |
| Normal, voiding dysfunction, not completed | 780164.50 | #VALUE! Unable to calculate CI | 0.991 |
| **Grade of operator** |  |  |  |
| Consultant | Reference |  |  |
| Other (Associate specialist, subspec trainee, speciality trainee, other, staff grade, FTSTA) | 0.53 | (0.21,1.30) | 0.167 |
| **Bladder injury** | 0.56 | (0.10,3.04) | 0.498 |

Table 25:Non-concomitant First surgery: Colposuspension: PGII

## Colposuspension: Change in SUI symptoms

| **Colposuspension** | **Populations:**  Non concomitant First surgery patients | | |
| --- | --- | --- | --- |
| **Secondary outcome:** |  |  |  |
| Change of SUI |  |  |  |
| No change or worse vs Cured or improved |  |  |  |
| **n=324** |  |  |  |
| **Multivariable analysis (accounting for Centre as a random effect)** | | |  |
| Number of imputations = 66 |  |  |  |
| **Characteristics** | **Odds ratio** | **95% CI** | **p-value** |
| **Age (decades)** | 0.47 | (0.25,0.87) | 0.017 |
| **BMI** | 0.94 | (0.76,1.15) | 0.536 |
| **PFMT** | 1.13 | (0.13,9.93) | 0.914 |
| **Pre operative urodynamic diagnosis** |  |  |  |
| USI, USI and voiding dysfunction | Reference |  |  |
| DOA, Mixed, mixed and voiding dysfunction | 7448532.00 | #VALUE! Unable to calculate CI | 0.994 |
| Normal, voiding dysfunction, not completed | 11000000.00 | #VALUE! Unable to calculate CI | 0.998 |
| **Grade of operator** |  |  |  |
| Consultant | Reference |  |  |
| Other (Associate specialist, subspec trainee, speciality trainee, other, staff grade, FTSTA) | 0.56 | (0.10,3.16) | 0.514 |
| **Bladder injury** | 0.40 | (0.04,4.10) | 0.438 |

Table 26:Non-concomitant First surgery: Colposuspension: SUI

## Colposuspension: Change in OAB symptoms: Patients with pre-operative symptoms

| **Colposuspension** | **Populations:**  Non concomitant First surgery patients | | | |
| --- | --- | --- | --- | --- |
| **Secondary outcome:** |  |  | |  |
| Change in OAB symptoms: Patients with pre-operative symptoms | | | |  |
| No change or worse vs Cured or improved |  |  | |  |
| **n=182** |  |  | |  |
| **Multivariable analysis (accounting for Centre as a random effect)** | | | |  |
| Number of imputations = 66 |  | |  |  |
| **Characteristics** | **Odds ratio** | | **95% CI** | **p-value** |
| **Age (decades)** | 1.20 | | (0.86,1.68) | 0.289 |
| **BMI** | 1.01 | | (0.92,1.11) | 0.813 |
| **PFMT** | 0.64 | | (0.21,2.03) | 0.453 |
| **Pre operative urodynamic diagnosis** |  | |  |  |
| USI, USI and voiding dysfunction | Reference | |  |  |
| DOA, Mixed, mixed and voiding dysfunction | 1.57 | | (0.70,3.50) | 0.274 |
| Normal, voiding dysfunction, not completed | 4987574.00 | | #VALUE! Unable to calculate CI | 0.989 |
| **Grade of operator** |  | |  |  |
| Consultant | Reference | |  |  |
| Other (Associate specialist, subspec trainee, speciality trainee, other, staff grade, FTSTA) | 0.57 | | (0.17,1.96) | 0.372 |
| **Bladder injury** | 1.29 | | (0.18,9.11) | 0.799 |

Table 27:Non-concomitant First surgery: Colposuspension: pre-op OAB

## Colposuspension: Change in OAB symptoms: Patients without pre-operative symptoms

| **Colposuspension** | **Populations:**  Non concomitant First surgery patients | | |
| --- | --- | --- | --- |
| **Secondary outcome:** |  |  |  |
| Change in OAB symptoms: Patients without pre-operative symptoms | | |  |
| Never present vs New symptoms |  |  |  |
| **n=134** |  |  |  |
| **Multivariable analysis (accounting for Centre as a random effect)** | | |  |
| Number of imputations = 66 |  |  |  |
| **Characteristics** | **Odds ratio** | **95% CI** | **p-value** |
| **Age (decades)** | 0.89 | (0.52,1.51) | 0.657 |
| **BMI** | 1.04 | (0.89,1.23) | 0.599 |
| **PFMT** | 0.88 | (0.21,3.69) | 0.857 |
| **Pre operative urodynamic diagnosis** |  |  |  |
| USI, USI and voiding dysfunction | Reference |  |  |
| DOA, Mixed, mixed and voiding dysfunction | 2.16 | (0.27,17.09) | 0.467 |
| Normal, voiding dysfunction, not completed | 1.93 | (0.12,31.03) | 0.641 |
| **Grade of operator** |  |  |  |
| Consultant | Reference |  |  |
| Other (Associate specialist, subspec trainee, speciality trainee, other, staff grade, FTSTA) | 1.45 | (0.35,5.98) | 0.606 |
| **Bladder injury** | 4.93 | (0.24,102.60) | 0.303 |

Table 28:Non-concomitant First surgery: Colposuspension: no pre-op OAB

## Colposuspension: Bladder injury

| **Colposuspension** | **Populations:**  Non concomitant First surgery patients | | |
| --- | --- | --- | --- |
| **Secondary outcome:** |  |  |  |
| Bladder injury |  |  |  |
| Yes vs No |  |  |  |
| **n=451** |  |  |  |
| **Multivariable analysis (accounting for Centre as a random effect)** | | |  |
| Number of imputations = 66 |  |  |  |
| **Characteristics** | **Odds ratio** | **95% CI** | **p-value** |
| **Age (decades)** | 1.67 | (1.03,2.71) | 0.036 |
| **BMI** | 1.01 | (0.89,1.15) | 0.867 |
| **Pre operative urodynamic diagnosis** |  |  |  |
| USI, USI and voiding dysfunction | Reference |  |  |
| DOA, Mixed, mixed and voiding dysfunction | 1.64 | (0.43,6.23) | 0.465 |
| Normal, voiding dysfunction, not completed | 0.00 | #VALUE! Unable to calculate CI | 0.993 |
| **Grade of operator** |  |  |  |
| Consultant | Reference |  |  |
| Other (Associate specialist, subspec trainee, speciality trainee, other, staff grade, FTSTA) | 1.00 | (0.21,4.74) | 0.998 |

Table 29:Non-concomitant First surgery: Colposuspension: bladder injury

## Colposuspension: Return to theatre for procedure-related event within 72 hours

| **Colposuspension** | **Populations:**  Non concomitant First surgery patients | | |
| --- | --- | --- | --- |
| **Secondary outcome:** |  |  |  |
| Return to theatre for procedure-related event within 72 hours | |  |  |
| Yes vs No |  |  |  |
| **n=365** |  |  |  |
| **Multivariable analysis (accounting for Centre as a random effect)** | | |  |
| Number of imputations = 44 |  |  |  |
| **Characteristics** | **Odds ratio** | **95% CI** | **p-value** |
| **Age (decades)** | 1.26 | (0.13,12.07) | 0.843 |
| **BMI** | 1.01 | (0.41,2.46) | 0.984 |
| **Pre operative urodynamic diagnosis** |  |  |  |
| USI, USI and voiding dysfunction | Reference |  |  |
| DOA, Mixed, mixed and voiding dysfunction | 0.00 | #VALUE! Unable to calculate CI | 0.994 |
| Normal, voiding dysfunction, not completed | 0.00 | #VALUE! Unable to calculate CI | 0.998 |
| **Grade of operator** |  |  |  |
| Consultant | Reference |  |  |
| Other (Associate specialist, subspec trainee, speciality trainee, other, staff grade, FTSTA) | 0.00 | #VALUE! Unable to calculate CI | 0.994 |
| **Bladder injury** | 0.00 | #VALUE! Unable to calculate CI | 0.998 |

Table 30:Non-concomitant First surgery: Colposuspension: return to theatre

## Colposuspension: Return to hospital within 30 days for procedure related event

| **Colposuspension** | **Populations:**  Non concomitant First surgery patients | | |
| --- | --- | --- | --- |
| **Secondary outcome:** |  |  |  |
| Return to hospital within 30 days for procedure related event | |  |  |
| Yes vs No |  |  |  |
| **n=303** |  |  |  |
| **Multivariable analysis (accounting for Centre as a random effect)** | | |  |
| Number of imputations = 66 |  |  |  |
| **Characteristics** | **Odds ratio** | **95% CI** | **p-value** |
| **Age (decades)** | 2.03 | (1.31,3.16) | 0.002 |
| **BMI** | 0.96 | (0.84,1.10) | 0.575 |
| **Pre operative urodynamic diagnosis** |  |  |  |
| USI, USI and voiding dysfunction | Reference |  |  |
| DOA, Mixed, mixed and voiding dysfunction | 0.50 | (0.10,2.46) | 0.394 |
| Normal, voiding dysfunction, not completed | 0.00 | #VALUE! Unable to calculate CI | 0.993 |
| **Grade of operator** |  |  |  |
| Consultant | Reference |  |  |
| Other (Associate specialist, subspec trainee, speciality trainee, other, staff grade, FTSTA) | 0.57 | (0.10,3.11) | 0.517 |
| **Bladder injury** | 1.56 | (0.15,16.09) | 0.711 |

Table 31:Non-concomitant First surgery: Colposuspension: return hospital

## Colposuspension: Readmitted to hospital within 30 days for procedure related event

| **Colposuspension** | | **Populations:**  Non concomitant First surgery patients | | |
| --- | --- | --- | --- | --- |
| **Secondary outcome:** | |  |  |  |
| Readmitted to hospital within 30 days for procedure related event | | |  |  |
| Yes vs No | |  |  |  |
| **n=360** | |  |  |  |
| **Multivariable analysis (accounting for Centre as a random effect)** | | | |  |
| Number of imputations = 65 |  | |  |  |
| **Characteristics** | **Odds ratio** | | **95% CI** | **p-value** |
| **Age (decades)** | 1.82 | | (1.12,2.95) | 0.016 |
| **BMI** | 1.10 | | (0.96,1.26) | 0.163 |
| **Pre operative urodynamic diagnosis** |  | |  |  |
| USI, USI and voiding dysfunction | Reference | |  |  |
| DOA, Mixed, mixed and voiding dysfunction | 0.27 | | (0.03,2.37) | 0.235 |
| Normal, voiding dysfunction, not completed | 0.00 | | #VALUE! Unable to calculate CI | 0.994 |
| **Grade of operator** |  | |  |  |
| Consultant | Reference | |  |  |
| Other (Associate specialist, subspec trainee, speciality trainee, other, staff grade, FTSTA) | 0.48 | | (0.08,2.78) | 0.411 |
| **Bladder injury** | 10.20 | | (1.56,66.76) | 0.015 |

Table 32:Non-concomitant First surgery: Colposuspension: readmit hospital

# Secondary population: All patients

# RPT

## RPT: PGII

| **RPT** | **Populations: All patients** | |  |
| --- | --- | --- | --- |
| **Primary outcome:** |  | |  |
| PGII |  |  |  |
| No change vs Better |  |  |  |
| (No change: A little better, No change, a little worse, much worse, very much worse | | | |
| Better: Very much better, much better) | | |  |
| **n=6,796** |  |  |  |
| **Multivariable analysis (accounting for Centre as a random effect)** | | | |
| Number of imputations = 0  The combination of variables and outcome did not use imputed data, therefore results are based on non-imputed data (i.e. original data) Error code: No imputations to calculate the between imputation variance | | | |
| **Characteristics** | **Odds ratio** | **95% CI** | **p-value** |
| **Age (decades)** | 0.83 | (0.78,0.89) | <0.001 |
| **BMI** | 0.95 | (0.94,0.97) | <0.001 |
| **PFMT** | 1.06 | (0.86,1.32) | 0.572 |
| **Pre operative urodynamic diagnosis** |  |  |  |
| USI, USI and voiding dysfunction | Reference |  |  |
| DOA, Mixed, mixed and voiding dysfunction | 0.52 | (0.42,0.63) | <0.001 |
| Normal, voiding dysfunction, not completed | 0.96 | (0.65,1.42) | 0.834 |
| **Grade of operator** |  |  |  |
| Consultant | Reference |  |  |
| Other (Associate specialist, subspec trainee, speciality trainee, other, staff grade, FTSTA) | 0.91 | (0.74,1.11) | 0.345 |
| **Bladder injury** | 0.61 | (0.41,0.92) | 0.018 |
| **Surgery type** |  |  |  |
| First | Reference |  |  |
| Repeat | 0.68 | (0.51,0.92) | 0.012 |

Table 33: All patients: RPT: PGII

## RPT: Change in SUI symptoms

| **RPT** | **Populations: All patients** | |  |
| --- | --- | --- | --- |
| **Secondary outcome:** |  |  |  |
| Change of SUI |  |  |  |
| No change or worse vs Cured or improved |  |  |  |
| **n=6,633** |  |  |  |
| **Multivariable analysis (accounting for Centre as a random effect)** | | |  |
| Number of imputations = 0  The combination of variables and outcome did not use imputed data, therefore results are based on non-imputed data (i.e. original data) Error code: No imputations to calculate the between imputation variance | | | |
| **Characteristics** | **Odds ratio** | **95% CI** | **p-value** |
| **Age (decades)** | 0.76 | (0.68,0.84) | <0.001 |
| **BMI** | 0.95 | (0.93,0.98) | <0.001 |
| **PFMT** | 0.79 | (0.56,1.12) | 0.185 |
| **Pre operative urodynamic diagnosis** |  |  |  |
| USI, USI and voiding dysfunction | Reference |  |  |
| DOA, Mixed, mixed and voiding dysfunction | 0.64 | (0.47,0.87) | 0.004 |
| Normal, voiding dysfunction, not completed | 1.04 | (0.55,1.98) | 0.893 |
| **Grade of operator** |  |  |  |
| Consultant | Reference |  |  |
| Other (Associate specialist, subspec trainee, speciality trainee, other, staff grade, FTSTA) | 0.95 | (0.68,1.33) | 0.753 |
| **Bladder injury** | 0.47 | (0.26,0.87) | 0.015 |
| **Surgery type** |  |  |  |
| First | Reference |  |  |
| Repeat | 0.55 | (0.36,0.83) | 0.004 |

Table 34: All patients: RPT: SUI

## RPT: Change in OAB symptoms: Patients with pre-operative symptoms

| **RPT** | **Populations: All patients** | | |  |
| --- | --- | --- | --- | --- |
| **Secondary outcome:** |  |  | |  |
| Change in OAB symptoms: Patients with pre-operative symptoms | | | |  |
| No change or worse vs Cured or improved |  | |  |  |
| **n=3,891** |  | |  |  |
| **Multivariable analysis (accounting for Centre as a random effect)** | | | |  |
| Number of imputations = 0  The combination of variables and outcome did not use imputed data, therefore results are based on non-imputed data (i.e. original data) Error code: No imputations to calculate the between imputation variance | | | | |
| **Characteristics** | **Odds ratio** | | **95% CI** | **p-value** |
| **Age (decades)** | 1.02 | | (0.96,1.09) | 0.451 |
| **BMI** | 0.99 | | (0.97,1.00) | 0.059 |
| **PFMT** | 0.92 | | (0.75,1.12) | 0.391 |
| **Pre operative urodynamic diagnosis** |  | |  |  |
| USI, USI and voiding dysfunction | Reference | |  |  |
| DOA, Mixed, mixed and voiding dysfunction | 0.91 | | (0.76,1.09) | 0.288 |
| Normal, voiding dysfunction, not completed | 0.93 | | (0.64,1.35) | 0.710 |
| **Grade of operator** |  | |  |  |
| Consultant | Reference | |  |  |
| Other (Associate specialist, subspec trainee, speciality trainee, other, staff grade, FTSTA) | 1.02 | | (0.85,1.22) | 0.856 |
| **Bladder injury** | 1.00 | | (0.67,1.49) | 0.984 |
| **Surgery type** |  | |  |  |
| First | Reference | |  |  |
| Repeat | 0.90 | | (0.68,1.20) | 0.474 |

Table 35: All patients: RPT: pre-op OAB

## RPT: Change in OAB symptoms: Patients without pre-operative symptoms

| **RPT** | **Populations: All patients** | |  |
| --- | --- | --- | --- |
| **Secondary outcome:** |  |  |  |
| Change in OAB symptoms: Patients without pre-operative symptoms | | |  |
| Never present vs New symptoms |  |  |  |
| **n=2,692** |  |  |  |
| **Multivariable analysis (accounting for Centre as a random effect)** |  |  |  |
| Number of imputations = 0  The combination of variables and outcome did not use imputed data, therefore results are based on non-imputed data (i.e. original data) Error code: No imputations to calculate the between imputation variance | | | |
| **Characteristics** | **Odds ratio** | **95% CI** | **p-value** |
| **Age (decades)** | 1.20 | (1.07,1.34) | 0.002 |
| **BMI** | 1.01 | (0.98,1.04) | 0.549 |
| **PFMT** | 1.06 | (0.70,1.61) | 0.775 |
| **Pre operative urodynamic diagnosis** |  |  |  |
| USI, USI and voiding dysfunction | Reference |  |  |
| DOA, Mixed, mixed and voiding dysfunction | 0.88 | (0.51,1.50) | 0.635 |
| Normal, voiding dysfunction, not completed | 0.96 | (0.57,1.61) | 0.874 |
| **Grade of operator** |  |  |  |
| Consultant | Reference |  |  |
| Other (Associate specialist, subspec trainee, speciality trainee, other, staff grade, FTSTA) | 1.49 | (1.09,2.05) | 0.013 |
| **Bladder injury** | 1.27 | (0.65,2.48) | 0.486 |
| **Surgery type** |  |  |  |
| First | Reference |  |  |
| Repeat | 0.68 | (0.33,1.38) | 0.285 |

Table 36: All patients: RPT: no pre-op OAB

## RPT: Bladder injury

| **RPT** | **Populations: All patients** | |  |
| --- | --- | --- | --- |
| **Secondary outcome:** |  |  |  |
| Bladder injury |  |  |  |
| Yes vs No |  |  |  |
| **n=12,558** |  |  |  |
| **Multivariable analysis (accounting for Centre as a random effect)** | | | |
| Number of imputations = 66 |  |  |  |
| **Characteristics** | **Odds ratio** | **95% CI** | **p-value** |
| **Age (decades)** | 1.06 | (0.97,1.15) | 0.230 |
| **BMI** | 0.92 | (0.90,0.95) | <0.001 |
| **Pre operative urodynamic diagnosis** |  |  |  |
| USI, USI and voiding dysfunction | Reference |  |  |
| DOA, Mixed, mixed and voiding dysfunction | 0.84 | (0.61,1.15) | 0.266 |
| Normal, voiding dysfunction, not completed | 0.62 | (0.36,1.07) | 0.084 |
| **Grade of operator** |  |  |  |
| Consultant | Reference |  |  |
| Other (Associate specialist, subspec trainee, speciality trainee, other, staff grade, FTSTA) | 4.00 | (3.20,5.00) | <0.001 |
| **Surgery type** |  |  |  |
| First | Reference |  |  |
| Repeat | 1.67 | (1.10,2.53) | 0.016 |

Table 37: All patients: RPT: bladder injury

## RPT: Return to theatre for procedure-related event within 72 hours

| **RPT** | **Populations: All patients** | |  |
| --- | --- | --- | --- |
| **Secondary outcome:** |  |  |  |
| Return to theatre for procedure-related event within 72 hours | |  |  |
| Yes vs No |  |  |  |
| **n=10,271** |  |  |  |
| **Multivariable analysis (accounting for Centre as a random effect)** | | | |
| Number of imputations = 66 |  |  |  |
| **Characteristics** | **Odds ratio** | **95% CI** | **p-value** |
| **Age (decades)** | 0.91 | (0.73,1.13) | 0.376 |
| **BMI** | 0.97 | (0.92,1.03) | 0.316 |
| **Pre operative urodynamic diagnosis** |  |  |  |
| USI, USI and voiding dysfunction | Reference |  |  |
| DOA, Mixed, mixed and voiding dysfunction | 0.75 | (0.36,1.55) | 0.432 |
| Normal, voiding dysfunction, not completed | 0.89 | (0.27,2.95) | 0.844 |
| **Grade of operator** |  |  |  |
| Consultant | Reference |  |  |
| Other (Associate specialist, subspec trainee, speciality trainee, other, staff grade, FTSTA) | 3.19 | (1.89,5.40) | <0.001 |
| **Bladder injury** | 3.03 | (1.42,6.48) | 0.004 |
| **Surgery type** |  |  |  |
| First | Reference |  |  |
| Repeat | 0.32 | (0.04,2.33) | 0.260 |

Table 38: All patients: RPT: Return to theatre

## RPT: Return to hospital within 30 days for procedure related event

| **RPT** | **Populations: All patients** | |  |
| --- | --- | --- | --- |
| **Secondary outcome:** |  |  |  |
| Return to hospital within 30 days for procedure related event | |  |  |
| Yes vs No |  |  |  |
| **n=4,060** |  |  |  |
| **Multivariable analysis (accounting for Centre as a random effect)** | | | |
| Number of imputations = 66 |  |  |  |
| **Characteristics** | **Odds ratio** | **95% CI** | **p-value** |
| **Age (decades)** | 1.02 | (0.92,1.13) | 0.727 |
| **BMI** | 1.00 | (0.97,1.04) | 0.826 |
| **Pre operative urodynamic diagnosis** |  |  |  |
| USI, USI and voiding dysfunction | Reference |  |  |
| DOA, Mixed, mixed and voiding dysfunction | 0.89 | (0.62,1.26) | 0.495 |
| Normal, voiding dysfunction, not completed | 1.43 | (0.87,2.35) | 0.163 |
| **Grade of operator** |  |  |  |
| Consultant | Reference |  |  |
| Other (Associate specialist, subspec trainee, speciality trainee, other, staff grade, FTSTA) | 1.23 | (0.89,1.68) | 0.206 |
| **Bladder injury** | 1.73 | (1.00,2.98) | 0.049 |
| **Surgery type** |  |  |  |
| First | Reference |  |  |
| Repeat | 1.07 | (0.60,1.90) | 0.817 |

Table 39: All patients: RPT: Return to hospital

## *RPT:* Readmitted to hospital within 30 days for procedure related event

| **RPT** | **Populations: All patients** | |  |
| --- | --- | --- | --- |
| **Secondary outcome:** |  |  |  |
| Readmitted to hospital within 30 days for procedure related event | |  |  |
| Yes vs No |  |  |  |
| **n=10,113** |  |  |  |
| **Multivariable analysis (accounting for Centre as a random effect)** | | | |
| Number of imputations = 66 |  |  |  |
| **Characteristics** | **Odds ratio** | **95% CI** | **p-value** |
| **Age (decades)** | 1.07 | (0.98,1.16) | 0.125 |
| **BMI** | 0.99 | (0.97,1.01) | 0.501 |
| **Pre operative urodynamic diagnosis** |  |  |  |
| USI, USI and voiding dysfunction | Reference |  |  |
| DOA, Mixed, mixed and voiding dysfunction | 1.26 | (0.97,1.63) | 0.086 |
| Normal, voiding dysfunction, not completed | 1.08 | (0.70,1.66) | 0.743 |
| **Grade of operator** |  |  |  |
| Consultant | Reference |  |  |
| Other (Associate specialist, subspec trainee, speciality trainee, other, staff grade, FTSTA) | 1.27 | (1.00,1.62) | 0.050 |
| **Bladder injury** | 1.79 | (1.16,2.78) | 0.009 |
| **Surgery type** |  |  |  |
| First | Reference |  |  |
| Repeat | 0.97 | (0.63,1.50) | 0.902 |

Table 40: All patients: RPT: readmitted to hospital

# PUB

## PUB: PGII

| **PUB** | **Populations:** All patients | |  |  |
| --- | --- | --- | --- | --- |
| **Primary outcome:** |  |  |  |  |
| PGII |  |  |  |  |
| No change vs Better |  |  |  |  |
| (No change: A little better, No change, a little worse, much worse, very much worse | | | | |
| Better: Very much better, much better) | | |  | |
| **n=676** |  |  |  |  |
| **Multivariable analysis (accounting for Centre as a random effect)** | | | | |
| Number of imputations = 0  The combination of variables and outcome did not use imputed data, therefore results are based on non-imputed data (i.e. original data) Error code: No imputations to calculate the between imputation variance | | | | |
| **Characteristics** | **Odds ratio** | **95% CI** | **p-value** |  |
| **Age (decades)** | 1.02 | (0.92,1.14) | 0.671 |  |
| **BMI** | 1.01 | (0.98,1.04) | 0.495 |  |
| **PFMT** | 1.11 | (0.67,1.83) | 0.696 |  |
| **Pre operative urodynamic diagnosis** |  |  |  |  |
| USI, USI and voiding dysfunction | Reference |  |  |  |
| DOA, Mixed, mixed and voiding dysfunction | 0.95 | (0.63,1.42) | 0.791 |  |
| Normal, voiding dysfunction, not completed | 0.53 | (0.26,1.07) | 0.077 |  |
| **Grade of operator** |  |  |  |  |
| Consultant | Reference |  |  |  |
| Other (Associate specialist, subspec trainee, speciality trainee, other, staff grade, FTSTA) | 1.00 | (0.59,1.69) | 0.995 |  |
| **Bladder injury** | Omitted |  |  |  |
| **Surgery type** |  |  |  |  |
| First | Reference |  |  |  |
| Repeat | 0.66 | (0.45,0.96) | 0.031 |  |

Table 41: All patients: PUB:PGI

## PUB: Change in SUI symptoms

| **PUB** | **Populations:** All patients | |  |
| --- | --- | --- | --- |
| **Secondary outcome:** |  |  |  |
| Change of SUI |  |  |  |
| No change or worse vs Cured or improved |  |  |  |
| **n=624** |  |  |  |
| **Multivariable analysis (accounting for Centre as a random effect)** | | | |
| Number of imputations = 0  The combination of variables and outcome did not use imputed data, therefore results are based on non-imputed data (i.e. original data) Error code: No imputations to calculate the between imputation variance | | | |
| **Characteristics** | **Odds ratio** | **95% CI** | **p-value** |
| **Age (decades)** | 0.99 | (0.87,1.13) | 0.892 |
| **BMI** | 1.01 | (0.98,1.05) | 0.454 |
| **PFMT** | 1.46 | (0.82,2.59) | 0.200 |
| **Pre operative urodynamic diagnosis** |  |  |  |
| USI, USI and voiding dysfunction | Reference |  |  |
| DOA, Mixed, mixed and voiding dysfunction | 0.73 | (0.46,1.17) | 0.188 |
| Normal, voiding dysfunction, not completed | 0.58 | (0.27,1.28) | 0.180 |
| **Grade of operator** |  |  |  |
| Consultant | Reference |  |  |
| Other (Associate specialist, subspec trainee, speciality trainee, other, staff grade, FTSTA) | 1.14 | (0.60,2.18) | 0.691 |
| **Bladder injury** | Omitted |  |  |
| **Surgery type** |  |  |  |
| First | Reference |  |  |
| Repeat | 0.58 | (0.37,0.91) | 0.018 |

Table 42: All patients: PUB:SUI

## *PUB :* Change in OAB symptoms: Patients with pre-operative symptoms

| **PUB** | **Populations:** All patients | |  |
| --- | --- | --- | --- |
| **Secondary outcome:** |  |  |  |
| Change in OAB symptoms: Patients with pre-operative symptoms | | | |
| No change or worse vs Cured or improved |  |  |  |
| **N = 395** |  |  |  |
| **Multivariable analysis (accounting for Centre as a random effect)** | | | |
| Number of imputations = 0  The combination of variables and outcome did not use imputed data, therefore results are based on non-imputed data (i.e. original data) Error code: No imputations to calculate the between imputation variance | | | |
| **Characteristics** | **Odds ratio** | **95% CI** | **p-value** |
| **Age (decades)** | 0.97 | (0.84,1.11) | 0.621 |
| **BMI** | 1.03 | (0.99,1.07) | 0.137 |
| **PFMT** | 1.01 | (0.52,1.94) | 0.986 |
| **Pre operative urodynamic diagnosis** |  |  |  |
| USI, USI and voiding dysfunction | Reference |  |  |
| DOA, Mixed, mixed and voiding dysfunction | 1.11 | (0.70,1.76) | 0.653 |
| Normal, voiding dysfunction, not completed | 1.41 | (0.58,3.43) | 0.454 |
| **Grade of operator** |  |  |  |
| Consultant | Reference |  |  |
| Other (Associate specialist, subspec trainee, speciality trainee, other, staff grade, FTSTA) | 0.49 | (0.23,1.03) | 0.061 |
| **Bladder injury** | Omitted |  |  |
| **Surgery type** |  |  |  |
| First | Reference |  |  |
| Repeat | 0.44 | (0.26,0.74) | 0.002 |

Table 43: All patients: PUB: pre-op OAB

## *PUB:* Change in OAB symptoms: Patients without pre-operative symptoms

| **PUB** | **Populations:** All patients | |  |
| --- | --- | --- | --- |
| **Secondary outcome:** |  |  |  |
| Change in OAB symptoms: Patients without pre-operative symptoms | | | |
| Never present vs New symptoms | **ERROR** |  |  |
| n = |  |  |  |
| **Multivariable analysis (accounting for Centre as a random effect)** |  | Number of imputations |  |
| **Characteristics** | **Odds ratio** | **95% CI** | **p-value** |
| **Age (decades)** | 0.00 | (0.00,0.00) | <0.001 |
| **BMI** | 0.00 | (0.00,0.00) | <0.001 |
| **PFMT** | 0.00 | (0.00,0.00) | <0.001 |
| **Pre operative urodynamic diagnosis** |  |  |  |
| USI, USI and voiding dysfunction | Reference |  |  |
| DOA, Mixed, mixed and voiding dysfunction | 0.00 | (0.00,0.00) | <0.001 |
| Normal, voiding dysfunction, not completed | 0.00 | (0.00,0.00) | <0.001 |
| **Grade of operator** |  |  |  |
| Consultant | Reference |  |  |
| Other (Associate specialist, subspec trainee, speciality trainee, other, staff grade, FTSTA) | 0.00 | (0.00,0.00) | <0.001 |
| **Bladder injury** | 0.00 | (0.00,0.00) | <0.001 |
| **Surgery type** |  |  |  |
| First | Reference |  |  |
| Repeat | 0.00 | (0.00,0.00) | <0.001 |

Table 44: All patients: PUB: No pre-op OAB

## PUB: Bladder injury

| **PUB** | **Populations:** All patients | | |  |
| --- | --- | --- | --- | --- |
| **Secondary outcome:** |  |  | |  |
| Bladder injury | **ERROR** |  | |  |
| Yes vs No |  |  | |  |
| **n=** |  |  | |  |
| **Multivariable analysis (accounting for Centre as a random effect)** | | | Number of imputations | |
| **Characteristics** | **Odds ratio** | **95% CI** | | **p-value** |
| **Age (decades)** | 0.00 | (0.00,0.00) | | <0.001 |
| **BMI** | 0.00 | (0.00,0.00) | | <0.001 |
| **Pre operative urodynamic diagnosis** |  |  | |  |
| USI, USI and voiding dysfunction | Reference |  | |  |
| DOA, Mixed, mixed and voiding dysfunction | 0.00 | (0.00,0.00) | | <0.001 |
| Normal, voiding dysfunction, not completed | 0.00 | (0.00,0.00) | | <0.001 |
| **Grade of operator** |  |  | |  |
| Consultant | Reference |  | |  |
| Other (Associate specialist, subspec trainee, speciality trainee, other, staff grade, FTSTA) | 0.00 | (0.00,0.00) | | <0.001 |
| **Surgery type** |  |  | |  |
| First | Reference |  | |  |
| Repeat | 0.00 | (0.00,0.00) | | <0.001 |

Table 45: All patients: PUB: bladder injury

## *PUB:* Return to theatre for procedure-related event within 72 hours

| **PUB** | **Populations:** All patients | |  |
| --- | --- | --- | --- |
| **Secondary outcome:** |  |  |  |
| Return to theatre for procedure-related event within 72 hours | |  |  |
| Yes vs No |  |  |  |
| **n= 1,665** |  |  |  |
| **Multivariable analysis (accounting for Centre as a random effect)** | | | |
| Number of imputations = 19 | | | |
| **Characteristics** | **Odds ratio** | **95% CI** | **p-value** |
| **Age (decades)** | 3.49 | (0.21,57.40) | 0.382 |
| **BMI** | 1.00 | (0.39,2.57) | 0.995 |
| **Pre operative urodynamic diagnosis** |  |  |  |
| USI, USI and voiding dysfunction | Reference |  |  |
| DOA, Mixed, mixed and voiding dysfunction | 1794827.00 | #VALUE! Unable to calculate a CI | 0.993 |
| Normal, voiding dysfunction, not completed | 0.13 | #VALUE! Unable to calculate a CI | 1.000 |
| **Grade of operator** |  |  |  |
| Consultant | Reference |  |  |
| Other (Associate specialist, subspec trainee, speciality trainee, other, staff grade, FTSTA) | 0.00 | #VALUE! Unable to calculate a CI | 0.995 |
| **Bladder injury** | Omitted |  |  |
| **Surgery type** |  |  |  |
| First | Reference |  |  |
| Repeat | 0.00 | #VALUE! Unable to calculate a CI | 0.994 |

Table 46: All patients: PUB: Return to theatre

## *PUB:* Return to hospital within 30 days for procedure related event

| **PUB** | **Populations:** All patients | |  |
| --- | --- | --- | --- |
| **Secondary outcome:** |  |  |  |
| Return to hospital within 30 days for procedure related event | |  |  |
| Yes vs No |  |  |  |
| **n=1,433** |  |  |  |
| **Multivariable analysis (accounting for Centre as a random effect)** | | | |
| Number of imputations = 66 |  |  |  |
| **Characteristics** | **Odds ratio** | **95% CI** | **p-value** |
| **Age (decades)** | 1.08 | (0.85,1.37) | 0.553 |
| **BMI** | 0.99 | (0.91,1.08) | 0.906 |
| **Pre operative urodynamic diagnosis** |  |  |  |
| USI, USI and voiding dysfunction | Reference |  |  |
| DOA, Mixed, mixed and voiding dysfunction | 1.53 | (0.64,3.69) | 0.339 |
| Normal, voiding dysfunction, not completed | 2.06 | (0.69,6.15) | 0.195 |
| **Grade of operator** |  |  |  |
| Consultant | Reference |  |  |
| Other (Associate specialist, subspec trainee, speciality trainee, other, staff grade, FTSTA) | 1.96 | (0.70,5.47) | 0.200 |
| **Bladder injury** | Omitted |  |  |
| **Surgery type** |  |  |  |
| First | Reference |  |  |
| Repeat | 0.66 | (0.24,1.80) | 0.416 |

Table 47: All patients: PUB: Return to hospital

## *PUB:* Readmitted to hospital within 30 days for procedure related event

| **PUB** | **Populations:** All patients | |  |
| --- | --- | --- | --- |
| **Secondary outcome:** |  |  |  |
| Readmitted to hospital within 30 days for procedure related event | |  |  |
| Yes vs No |  |  |  |
| **n=1,646** |  |  |  |
| **Multivariable analysis (accounting for Centre as a random effect)** | | | |
| Number of imputations = 65 |  |  |  |
| **Characteristics** | **Odds ratio** | **95% CI** | **p-value** |
| **Age (decades)** | 0.25 | (1.76,0.00) | 0.746 |
| **BMI** | 0.94 | (0.79,1.13) | 0.519 |
| **Pre operative urodynamic diagnosis** |  |  |  |
| USI, USI and voiding dysfunction | Reference |  |  |
| DOA, Mixed, mixed and voiding dysfunction | 2.52 | (0.69,9.19) | 0.162 |
| Normal, voiding dysfunction, not completed | 0.00 | #VALUE! Unable to calculate a CI | 0.994 |
| **Grade of operator** |  |  |  |
| Consultant | Reference |  |  |
| Other (Associate specialist, subspec trainee, speciality trainee, other, staff grade, FTSTA) | 3.53 | (0.89,13.95) | 0.072 |
| **Bladder injury** | Omitted |  |  |
| **Surgery type** |  |  |  |
| First | Reference |  |  |
| Repeat | 1.54 | (0.39,6.08) | 0.538 |

Table 48: All patients: PUB: readmitted to hospital

# AFS

## AFS: PGII

| **AFS** | **Populations:** All patients | |  |
| --- | --- | --- | --- |
| **Primary outcome:** |  |  |  |
| PGII |  |  |  |
| No change vs Better |  |  |  |
| (No change: A little better, No change, a little worse, much worse, very much worse | | | |
| Better: Very much better, much better) | | |  |
| **n=54** |  |  |  |
| **Multivariable analysis (accounting for Centre as a random effect)** | | | |
| Number of imputations = 0  The combination of variables and outcome did not use imputed data, therefore results are based on non-imputed data (i.e. original data) Error code: No imputations to calculate the between imputation variance | | | |
| **Characteristics** | **Odds ratio** | **95% CI** | **p-value** |
| **Age (decades)** | 1.25 | (0.38,4.07) | 0.711 |
| **BMI** | 0.78 | (0.59,1.02) | 0.072 |
| **PFMT** | 0.41 | (0.02,9.63) | 0.577 |
| **Pre operative urodynamic diagnosis** |  |  |  |
| USI, USI and voiding dysfunction | Reference |  |  |
| DOA, Mixed, mixed and voiding dysfunction | 8155642.00 | #VALUE! Unable to calculate a CI | 0.995 |
| Normal, voiding dysfunction, not completed | 0.27 | (0.01,6.59) | 0.423 |
| **Grade of operator** |  |  |  |
| Consultant | Reference |  |  |
| Other (Associate specialist, subspec trainee, speciality trainee, other, staff grade, FTSTA) | 0.12 | (0.00,3.26) | 0.205 |
| **Bladder injury** | 0.05 | (0.00,2.60) | 0.137 |
| **Surgery type** |  |  |  |
| First | Reference |  |  |
| Repeat | 0.37 | (0.03,4.84) | 0.451 |

Table 49: All patients: AFS: PGII

## *AFS:* Change of SUI symptoms

| **AFS** | **Populations:** All patients | |  | |
| --- | --- | --- | --- | --- |
| **Secondary outcome:** |  |  |  | |
| Change of SUI |  |  |  | |
| No change or worse vs Cured or improved |  |  |  | |
| **n=50** |  |  |  | |
| **Multivariable analysis (accounting for Centre as a random effect)** | | | |  |
| Number of imputations = 0  The combination of variables and outcome did not use imputed data, therefore results are based on non-imputed data (i.e. original data) Error code: No imputations to calculate the between imputation variance | | | | |
| **Characteristics** | **Odds ratio** | **95% CI** | **p-value** | |
| **Age (decades)** | 0.70 | (0.16,2.94) | 0.622 | |
| **BMI** | 0.69 | (0.44,1.09) | 0.112 | |
| **PFMT** | 0.00 | #VALUE! Unable to calculate a CI | 0.995 | |
| **Pre operative urodynamic diagnosis** |  |  |  | |
| USI, USI and voiding dysfunction | Reference |  |  | |
| DOA, Mixed, mixed and voiding dysfunction | 182000000.00 | #VALUE! Unable to calculate a CI | 0.998 | |
| Normal, voiding dysfunction, not completed | 0.23 | (0.00,10.93) | 0.456 | |
| **Grade of operator** |  |  |  | |
| Consultant | Reference |  |  | |
| Other (Associate specialist, subspec trainee, speciality trainee, other, staff grade, FTSTA) | 0.01 | (0.00,2.56) | 0.100 | |
| **Bladder injury** | 245000000.00 | #VALUE! Unable to calculate a CI | 0.999 | |
| **Surgery type** |  |  |  | |
| First | Reference |  |  | |
| Repeat | 0.31 | (0.01,7.64) | 0.476 | |

Table 50: All patients: AFS: SUI

## *AFS:* Change in OAB symptoms: Patients with pre-operative symptoms

| **AFS** | **Populations:** All patients | |  |
| --- | --- | --- | --- |
| **Secondary outcome:** |  |  |  |
| Change in OAB symptoms: Patients with pre-operative symptoms | | | |
| No change or worse vs Cured or improved |  |  |  |
| **n=28** |  |  |  |
| **Multivariable analysis (accounting for Centre as a random effect)** | | | |
| Number of imputations = 0  The combination of variables and outcome did not use imputed data, therefore results are based on non-imputed data (i.e. original data) Error code: No imputations to calculate the between imputation variance | | | |
| **Characteristics** | **Odds ratio** | **95% CI** | **p-value** |
| **Age (decades)** | 1.74 | (0.29,10.58) | 0.546 |
| **BMI** | 0.90 | (0.67,1.21) | 0.471 |
| **PFMT** | 0.47 | (0.01,16.15) | 0.674 |
| **Pre operative urodynamic diagnosis** |  |  |  |
| USI, USI and voiding dysfunction | Reference |  |  |
| DOA, Mixed, mixed and voiding dysfunction | 0.69 | (0.03,15.95) | 0.817 |
| Normal, voiding dysfunction, not completed | 12.44 | (0.21,727.75) | 0.225 |
| **Grade of operator** |  |  |  |
| Consultant | Reference |  |  |
| Other (Associate specialist, subspec trainee, speciality trainee, other, staff grade, FTSTA) | 3.18 | (0.02,446.24) | 0.646 |
| **Bladder injury** | 26800000.00 | #VALUE! Unable to calculate a CI | 0.995 |
| **Surgery type** |  |  |  |
| First | Reference |  |  |
| Repeat | 2.27 | (0.09,55.45) | 0.614 |

Table 51: All patients: AFS: pre-op OAB

## AFS: Change in OAB symptoms: Patients without pre-operative symptoms

| **AFS** | **Populations:** All patients | |  |
| --- | --- | --- | --- |
| **Secondary outcome:** |  |  |  |
| Change in OAB symptoms: Patients without pre-operative symptoms | | |  |
| Never present vs New symptoms |  |  |  |
|  | **ERROR** |  |  |
| **n=** |  |  |  |
| **Multivariable analysis (accounting for Centre as a random effect)** | | | |
| Number of imputations |  |  |  |
| **Characteristics** | **Odds ratio** | **95% CI** | **p-value** |
| **Age (decades)** | 0.00 | (0.00,0.00) | <0.001 |
| **BMI** | 0.00 | (0.00,0.00) | <0.001 |
| **PFMT** | 0.00 | (0.00,0.00) | <0.001 |
| **Pre operative urodynamic diagnosis** |  |  |  |
| USI, USI and voiding dysfunction | Reference |  |  |
| DOA, Mixed, mixed and voiding dysfunction | 0.00 | (0.00,0.00) | <0.001 |
| Normal, voiding dysfunction, not completed | 0.00 | (0.00,0.00) | <0.001 |
| **Grade of operator** |  |  |  |
| Consultant | Reference |  |  |
| Other (Associate specialist, subspec trainee, speciality trainee, other, staff grade, FTSTA) | 0.00 | (0.00,0.00) | <0.001 |
| **Bladder injury** | 0.00 | (0.00,0.00) | <0.001 |
| **Surgery type** |  |  |  |
| First | Reference |  |  |
| Repeat | 0.00 | (0.00,0.00) | <0.001 |

Table 52: All patients: AFS: No pre-op OAB

## *AFS:* Bladder injury

| **AFS** | **Populations:** All patients | |  |
| --- | --- | --- | --- |
| **Secondary outcome:** |  |  |  |
| Bladder injury |  |  |  |
| Yes vs No |  |  |  |
| **n=233** |  |  |  |
| **Multivariable analysis (accounting for Centre as a random effect)** | | | |
| Number of imputations = 50 |  |  |  |
| **Characteristics** | **Odds ratio** | **95% CI** | **p-value** |
| **Age (decades)** | 0.98 | (0.56,1.73) | 0.942 |
| **BMI** | 0.90 | (0.75,1.09) | 0.300 |
| **Pre operative urodynamic diagnosis** |  |  |  |
| USI, USI and voiding dysfunction | Reference |  |  |
| DOA, Mixed, mixed and voiding dysfunction | 0.00 | #VALUE! Unable to calculate a CI | 0.993 |
| Normal, voiding dysfunction, not completed | 0.00 | #VALUE! Unable to calculate a CI | 0.995 |
| **Grade of operator** |  |  |  |
| Consultant | Reference |  |  |
| Other (Associate specialist, subspec trainee, speciality trainee, other, staff grade, FTSTA) | 1.77 | (0.30,10.60) | 0.533 |
| **Surgery type** |  |  |  |
| First | Reference |  |  |
| Repeat | 2.01 | (0.40,10.04) | 0.394 |

Table 53: All patients: AFS: bladder injury

## *AFS: R*eturn to theatre for procedure-related event within 72 hours

| **AFS** | **Populations:** All patients | |  | |
| --- | --- | --- | --- | --- |
| **Secondary outcome:** |  |  |  | |
| Return to theatre for procedure-related event within 72 hours | |  | |  |
| Yes vs No |  |  |  | |
| **n=159** |  |  |  | |
| **Multivariable analysis (accounting for Centre as a random effect)** | | | | |
| Number of imputations = 4 |  |  |  | |
| **Characteristics** | **Odds ratio** | **95% CI** | **p-value** | |
| **Age (decades)** | 0.14 | (0.00,455.36) | 0.635 | |
| **BMI** | 0.31 | (0.00,43.82) | 0.639 | |
| **Pre operative urodynamic diagnosis** |  |  |  | |
| USI, USI and voiding dysfunction | Reference |  |  | |
| DOA, Mixed, mixed and voiding dysfunction | 0.00 | #VALUE! Unable to calculate a CI | 0.996 | |
| Normal, voiding dysfunction, not completed | 0.00 | #VALUE! Unable to calculate a CI | 0.998 | |
| **Grade of operator** |  |  |  | |
| Consultant | Reference |  |  | |
| Other (Associate specialist, subspec trainee, speciality trainee, other, staff grade, FTSTA) | 0.00 | #VALUE! Unable to calculate a CI | 0.995 | |
| **Bladder injury** | 0.00 | #VALUE! Unable to calculate a CI | 0.999 | |
| **Surgery type** |  |  |  | |
| First | Reference |  |  | |
| Repeat | 355329.40 | #VALUE! Unable to calculate a CI | 0.988 | |

Table 54: All patients: AFS: Return to theatre

## *AFS:* Return to hospital within 30 days for procedure related event

| **AFS** | **Populations:** All patients | | |  |
| --- | --- | --- | --- | --- |
|  |  |  | |  |
| **Secondary outcome:** |  |  | |  |
| Return to hospital within 30 days for procedure related event | | |  |  |
| Yes vs No |  |  | |  |
| **n=140** |  |  | |  |
| **Multivariable analysis (accounting for Centre as a random effect)** | | | | |
| Number of imputations = 66 |  |  | |  |
| **Characteristics** | **Odds ratio** | **95% CI** | | **p-value** |
| **Age (decades)** | 1.01 | (0.65,1.55) | | 0.982 |
| **BMI** | 0.95 | (0.84,1.08) | | 0.449 |
| **Pre operative urodynamic diagnosis** |  |  | |  |
| USI, USI and voiding dysfunction | Reference |  | |  |
| DOA, Mixed, mixed and voiding dysfunction | 1.22 | (0.39,3.84) | | 0.733 |
| Normal, voiding dysfunction, not completed | 0.65 | (0.07,5.71) | | 0.695 |
| **Grade of operator** |  |  | |  |
| Consultant | Reference |  | |  |
| Other (Associate specialist, subspec trainee, speciality trainee, other, staff grade, FTSTA) | 0.36 | (0.07,1.77) | | 0.209 |
| **Bladder injury** | 6.06 | (0.66,55.56) | | 0.111 |
| **Surgery type** |  |  | |  |
| First | Reference |  | |  |
| Repeat | 0.98 | (0.38,2.54) | | 0.962 |

Table 55: All patients: AFS: Return to hospital

## *AFS:* Readmitted to hospital within 30 days for procedure related event

| **AFS** | **Populations:** All patients | | |  | |
| --- | --- | --- | --- | --- | --- |
| **Secondary outcome:** |  |  | |  | |
| Readmitted to hospital within 30 days for procedure related event | | |  | |  |
| Yes vs No |  |  | |  | |
| **n=159** |  |  | |  | |
| **Multivariable analysis (accounting for Centre as a random effect)** | | | | | |
| Number of imputations = 66 |  |  | |  | |
| **Characteristics** | **Odds ratio** | **95% CI** | | **p-value** | |
| **Age (decades)** | 1.55 | (1.00,2.40) | | 0.048 | |
| **BMI** | 1.02 | (0.89,1.17) | | 0.790 | |
| **Pre operative urodynamic diagnosis** |  |  | |  | |
| USI, USI and voiding dysfunction | Reference |  | |  | |
| DOA, Mixed, mixed and voiding dysfunction | 1.98 | (0.58,6.81) | | 0.276 | |
| Normal, voiding dysfunction, not completed | 1.32 | (0.14,12.23) | | 0.806 | |
| **Grade of operator** |  |  | |  | |
| Consultant | Reference |  | |  | |
| Other (Associate specialist, subspec trainee, speciality trainee, other, staff grade, FTSTA) | 0.64 | (0.13,3.25) | | 0.595 | |
| **Bladder injury** | 4.74 | (0.38,59.90) | | 0.229 | |
| **Surgery type** |  |  | |  | |
| First | Reference |  | |  | |
| Repeat | 0.35 | (0.10,1.16) | | 0.086 | |

Table 56: All patients: AFS: Readmitted to hospital

# Colposuspension

## Colposuspension: PGII

| **Colposuspension** | **Populations:** All patients | | | | | | |
| --- | --- | --- | --- | --- | --- | --- | --- |
| **Primary outcome:** |  | | | |  | | |
| PGII |  | | | |  | | |
| No change vs Better |  | | | |  | | |
| (No change: A little better, No change, a little worse, much worse, very much worse | | | | | | | |
| Better: Very much better, much better) |  | | | | | | |
| **n=377** |  | |  | | |  | |
| **Multivariable analysis (accounting for Centre as a random effect)** | | | | | | | |
| Number of imputations = 0  The combination of variables and outcome did not use imputed data, therefore results are based on non-imputed data (i.e. original data) Error code: No imputations to calculate the between imputation variance | | | | | | | |
| **Characteristics** | | **Odds ratio** | | **95% CI** | | | **p-value** |
| **Age (decades)** | | 0.83 | | (0.63,1.09) | | | 0.176 |
| **BMI** | | 1.01 | | (0.95,1.09) | | | 0.697 |
| **PFMT** | | 1.11 | | (0.49,2.50) | | | 0.810 |
| **Pre operative urodynamic diagnosis** | |  | |  | | |  |
| USI, USI and voiding dysfunction | | Reference | |  | | |  |
| DOA, Mixed, mixed and voiding dysfunction | | 1.19 | | (0.43,3.29) | | | 0.739 |
| Normal, voiding dysfunction, not completed | | 0.88 | | (0.09,8.58) | | | 0.910 |
| **Grade of operator** | |  | |  | | |  |
| Consultant | | Reference | |  | | |  |
| Other (Associate specialist, subspec trainee, speciality trainee, other, staff grade, FTSTA) | | 1.22 | | (0.44,3.39) | | | 0.707 |
| **Bladder injury** | | 0.63 | | (0.12,3.28) | | | 0.580 |
| **Surgery type** | |  | |  | | |  |
| First | | Reference | |  | | |  |
| Repeat | | 0.43 | | (0.22,0.86) | | | 0.016 |

Table 57: All patients: Colposuspension: PGII

## Colposuspension: Change in SUI symptoms

| **Colposuspension** | **Populations:** All patients | | |
| --- | --- | --- | --- |
| **Secondary outcome:** |  |  |  |
| Change of SUI |  |  |  |
| No change or worse vs Cured or improved |  |  |  |
| **n=360** |  |  |  |
| **Multivariable analysis (accounting for Centre as a random effect)** | | | |
| Number of imputations = 0  The combination of variables and outcome did not use imputed data, therefore results are based on non-imputed data (i.e. original data) Error code: No imputations to calculate the between imputation variance | | | |
| **Characteristics** | **Odds ratio** | **95% CI** | **p-value** |
| **Age (decades)** | 0.70 | (0.46,1.07) | 0.102 |
| **BMI** | 1.00 | (0.90,1.11) | 0.938 |
| **PFMT** | 0.70 | (0.18,2.77) | 0.617 |
| **Pre operative urodynamic diagnosis** |  |  |  |
| USI, USI and voiding dysfunction | Reference |  |  |
| DOA, Mixed, mixed and voiding dysfunction | 0.49 | (0.12,1.97) | 0.318 |
| Normal, voiding dysfunction, not completed | 0.20 | (0.02,2.07) | 0.177 |
| **Grade of operator** |  |  |  |
| Consultant | Reference |  |  |
| Other (Associate specialist, subspec trainee, speciality trainee, other, staff grade, FTSTA) | 0.31 | (0.09,1.12) | 0.074 |
| **Bladder injury** | 600843.00 | #VALUE! Unable to calculate CI | 0.991 |
| **Surgery type** |  |  |  |
| First | Reference |  |  |
| Repeat | 0.23 | (0.08,0.66) | 0.006 |

Table 58: All patients: Colposuspension: SUI

## Colposuspension: Change in OAB symptoms: Patients with pre-operative symptoms

| **Colposuspension** | **Populations:** All patients | |  |
| --- | --- | --- | --- |
| **Secondary outcome:** |  |  |  |
| Change in OAB symptoms: Patients with pre-operative symptoms | | | |
| No change or worse vs Cured or improved |  |  |  |
| **n=188** |  |  |  |
| **Multivariable analysis (accounting for Centre as a random effect)** | | | |
| Number of imputations = 0  The combination of variables and outcome did not use imputed data, therefore results are based on non-imputed data (i.e. original data) Error code: No imputations to calculate the between imputation variance | | | |
| **Characteristics** | **Odds ratio** | **95% CI** | **p-value** |
| **Age (decades)** | 1.31 | (0.96,1.78) | 0.092 |
| **BMI** | 0.99 | (0.93,1.06) | 0.808 |
| **PFMT** | 0.70 | (0.29,1.72) | 0.441 |
| **Pre operative urodynamic diagnosis** |  |  |  |
| USI, USI and voiding dysfunction | Reference |  |  |
| DOA, Mixed, mixed and voiding dysfunction | 0.85 | (0.37,1.92) | 0.691 |
| Normal, voiding dysfunction, not completed | 1.77 | (0.25,12.53) | 0.570 |
| **Grade of operator** |  |  |  |
| Consultant | Reference |  |  |
| Other (Associate specialist, subspec trainee, speciality trainee, other, staff grade, FTSTA) | 1.00 | (0.32,3.15) | 0.998 |
| **Bladder injury** | 0.98 | (0.16,6.02) | 0.985 |
| **Surgery type** |  |  |  |
| First | Reference |  |  |
| Repeat | 0.69 | (0.31,1.52) | 0.356 |

Table 59: All patients: Colposuspension: pre-op OAB

## Colposuspension: Change in OAB symptoms: Patients without pre-operative symptoms

| **Colposuspension** | **Populations:** All patients | | | |  |
| --- | --- | --- | --- | --- | --- |
| **Secondary outcome:** | **ERROR** | |  | |  |
| Change in OAB symptoms: Patients without pre-operative symptoms | | | | |  |
| Never present vs New symptoms |  | |  | |  |
| **n=** |  | |  | |  |
| **Multivariable analysis (accounting for Centre as a random effect)** | | | | | |
| Number of imputations |  |  | |  | |
| **Characteristics** | **Odds ratio** | **95% CI** | | **p-value** | |
| **Age (decades)** | 0.00 | (0.00,0.00) | | <0.001 | |
| **BMI** | 0.00 | (0.00,0.00) | | <0.001 | |
| **PFMT** | 0.00 | (0.00,0.00) | | <0.001 | |
| **Pre operative urodynamic diagnosis** |  |  | |  | |
| USI, USI and voiding dysfunction | Reference |  | |  | |
| DOA, Mixed, mixed and voiding dysfunction | 0.00 | (0.00,0.00) | | <0.001 | |
| Normal, voiding dysfunction, not completed | 0.00 | (0.00,0.00) | | <0.001 | |
| **Grade of operator** |  |  | |  | |
| Consultant | Reference |  | |  | |
| Other (Associate specialist, subspec trainee, speciality trainee, other, staff grade, FTSTA) | 0.00 | (0.00,0.00) | | <0.001 | |
| **Bladder injury** | 0.00 | (0.00,0.00) | | <0.001 | |
| **Surgery type** |  |  | |  | |
| First | Reference |  | |  | |
| Repeat | 0.00 | (0.00,0.00) | | <0.001 | |

Table 60: All patients: Colposuspension: no pre-op OAB

## Colposuspension: Bladder injury

| **Colposuspension** | **Populations:** All patients | |  |
| --- | --- | --- | --- |
| **Secondary outcome:** |  |  |  |
| Bladder injury |  |  |  |
| Yes vs No |  |  |  |
| **n=906** |  |  |  |
| **Multivariable analysis (accounting for Centre as a random effect)** | | | |
| Number of imputations = 65 |  |  |  |
|  |  |  |  |
| **Characteristics** | **Odds ratio** | **95% CI** | **p-value** |
| **Age (decades)** | 1.32 | (0.96,1.83) | 0.092 |
| **BMI** | 1.01 | (0.92,1.11) | 0.810 |
| **Pre operative urodynamic diagnosis** |  |  |  |
| USI, USI and voiding dysfunction | Reference |  |  |
| DOA, Mixed, mixed and voiding dysfunction | 0.92 | (0.27,3.21) | 0.902 |
| Normal, voiding dysfunction, not completed | 0.00 | #VALUE! Unable to calculate a CI | 0.992 |
| **Grade of operator** |  |  |  |
| Consultant | Reference |  |  |
| Other (Associate specialist, subspec trainee, speciality trainee, other, staff grade, FTSTA) | 0.88 | (0.25,3.10) | 0.838 |
| **Surgery type** |  |  |  |
| First | Reference |  |  |
| Repeat | 1.49 | (0.58,3.82) | 0.404 |

Table 61: All patients: Colposuspension: Bladder injury

## Colposuspension: Return to theatre for procedure-related event within 72 hours

| **Colposuspension** | **Populations:** All patients | |  |
| --- | --- | --- | --- |
| **Secondary outcome:** |  |  |  |
| Return to theatre for procedure-related event within 72 hours | |  |  |
| Yes vs No |  |  |  |
| **n=738** |  |  |  |
| **Multivariable analysis (accounting for Centre as a random effect)** | | | |
| Number of imputations = 26 |  |  |  |
| **Characteristics** | **Odds ratio** | **95% CI** | **p-value** |
| **Age (decades)** | 0.67 | (0.25,1.80) | 0.429 |
| **BMI** | 1.00 | (0.72,1.38) | 0.982 |
| **Pre operative urodynamic diagnosis** |  |  |  |
| USI, USI and voiding dysfunction | Reference |  |  |
| DOA, Mixed, mixed and voiding dysfunction | 0.00 | #VALUE! Unable to calculate a CI | 0.990 |
| Normal, voiding dysfunction, not completed | 0.00 | #VALUE! Unable to calculate a CI | 0.997 |
| **Grade of operator** |  |  |  |
| Consultant | Reference |  |  |
| Other (Associate specialist, subspec trainee, speciality trainee, other, staff grade, FTSTA) | 0.00 | #VALUE! Unable to calculate a CI | 0.991 |
| **Bladder injury** | 19.19 | (1.47,251.25) | 0.024 |
| **Surgery type** |  |  |  |
| First | Reference |  |  |
| Repeat | 3.53 | (0.47,26.71) | 0.223 |

Table 62: All patients: Colposuspension: Return to theatre

## Colposuspension: Return to theatre for procedure-related event within 72 hours

| **Colposuspension** | **Populations:** All patients | |  |
| --- | --- | --- | --- |
| **Secondary outcome:** |  |  |  |
| Return to theatre for procedure-related event within 72 hours | |  |  |
| Yes vs No |  |  |  |
| **n=548** |  |  |  |
| **Multivariable analysis (accounting for Centre as a random effect)** | | |  |
| Number of imputations = 66 |  |  | 66 |
| **Characteristics** | **Odds ratio** | **95% CI** | **p-value** |
| **Age (decades)** | 1.43 | (1.09,1.88) | 0.011 |
| **BMI** | 1.00 | (0.92,1.08) | 0.968 |
| **Pre operative urodynamic diagnosis** |  |  |  |
| USI, USI and voiding dysfunction | Reference |  |  |
| DOA, Mixed, mixed and voiding dysfunction | 1.07 | (0.45,2.57) | 0.875 |
| Normal, voiding dysfunction, not completed | 0.00 | #VALUE! Unable to calculate a CI | 0.987 |
| **Grade of operator** |  |  |  |
| Consultant | Reference |  |  |
| Other (Associate specialist, subspec trainee, speciality trainee, other, staff grade, FTSTA) | 0.88 | (0.35,2.22) | 0.784 |
| **Bladder injury** | 1.14 | (0.20,6.63) | 0.882 |
| **Surgery type** |  |  |  |
| First | Reference |  |  |
| Repeat | 1.53 | (0.74,3.15) | 0.247 |

Table 63: All patients: Colposuspension: Return to hospital

## Colposuspension: Readmitted to hospital within 30 days for procedure related event

| **Colposuspension** | **Populations:** All patients | |  |
| --- | --- | --- | --- |
| **Secondary outcome:** |  |  |  |
| Readmitted to hospital within 30 days for procedure related event | |  |  |
| Yes vs No |  |  |  |
| **n=726** |  |  |  |
| **Multivariable analysis (accounting for Centre as a random effect)** | | | |
| Number of imputations = 66 |  |  |  |
| **Characteristics** | **Odds ratio** | **95% CI** | **P-value** |
| **Age (decades)** | 1.26 | (0.96,1.66) | 0.094 |
| **BMI** | 1.06 | (0.98,1.15) | 0.128 |
| **Pre operative urodynamic diagnosis** |  |  |  |
| USI, USI and voiding dysfunction | Reference |  |  |
| DOA, Mixed, mixed and voiding dysfunction | 0.67 | (0.22,2.01) | 0.470 |
| Normal, voiding dysfunction, not completed | 0.93 | (0.11,7.63) | 0.944 |
| **Grade of operator** |  |  |  |
| Consultant | Reference |  |  |
| Other (Associate specialist, subspec trainee, speciality trainee, other, staff grade, FTSTA) | 1.04 | (0.41,2.62) | 0.933 |
| **Bladder injury** | 6.49 | (1.95,21.55) | 0.002 |
| **Surgery type** |  |  |  |
| First | Reference |  |  |
| Repeat | 0.81 | (0.36,1.85) | 0.623 |

Table 64: All patients: Colposuspension: Readmitted to hospital
